# Hypothalamic and sex-related hormones in migraine

**DOI:** 10.1186/s10194-026-02289-z

**Published:** 2026-02-12

**Authors:** Karin Warfvinge, Jacob C. A. Edvinsson, Aida Maddahi, Lars Edvinsson

**Affiliations:** Division of Experimental Vascular Research, Department of Clinical Sciences, Lund University, Lund, Sweden

## Abstract

The hypothalamus and sex hormones are closely linked to primary headache disorders. Migraine is the most prevalent disabling neurological disorder, affecting up to 15% of the global population, with enormous socioeconomic and personal impacts. It ranks second among years lived with disability (YLD), with women being the most affected, experiencing a threefold higher prevalence than men. Women report more severe migraine symptoms during periods of sex hormone fluctuations, particularly at puberty, during pregnancy, and around perimenopause. The reason for this sex-based difference remains unclear. However, it is apparent that dynamic fluctuations in female sex hormone levels (oestrogen, progesterone) have a key influence on the susceptibility to attacks in women. A fall in plasma levels of oestrogen, progesterone, and hypothalamic oxytocin may trigger migraine attacks, whereas higher levels appear to be protective. Circulating hormones show dynamic alterations, and, more importantly, their receptors are expressed in the trigeminovascular system (TGVS). Notably, other hormones, such as testosterone and vasopressin (AVP), are less extensively studied but are also highlighted in this review. The underlying mechanisms of their effects on migraine targets have yet to be explained. This review highlights the interplay between sex/reproductive hormones and hypothalamic hormones and their receptors in migraine-related neural structures, both in the central nervous system and in the TGVS. We have included the hypothalamic hormones oxytocin and the molecularly related AVP due to the significant drop in oxytocin levels at the onset of menstruation, as well as its role in parturition and lactation. Recent data have revealed expression of oxytocin receptors in the TGVS. Oestrogen, acting through multiple receptor subtypes, influences expression levels of oxytocin and its receptor. Recent data have demonstrated a close transcriptional relationship between oestrogen and calcitonin gene-related peptide (CGRP), as well as the receptor component “receptor activity-modifying protein 1” (RAMP1), in migraine pathology and current successful treatments.

## Introduction

Primary headaches comprise a group of disorders amounting to nearly 300 subtypes, as defined by the International Classification of Headache Disorders (ICHD-3) [1]. Migraine is clinically well characterised by a distinctive sequence of symptoms, including unilateral throbbing or pulsating headache, accompanied by nausea, vomiting, and sensitivity to sound (phonophobia) and light (photophobia) [1]. Migraine is highly prevalent, affecting nearly 15% of the global population, and ranks among the most burdensome disorders, with profound personal and socioeconomic consequences. Women are disproportionately affected, with up to three times higher prevalence than men [2, 3]. Furthermore, women report longer attack duration, increased risk of recurrence, greater disability, and extended recovery periods [4]. According to the Global Burden of Disease Study 2017 [3] migraine is the leading cause of years lived with disability (YLD) among women of reproductive age (15–49 years) [5].

Epidemiological research has revealed an association between migraine attacks and various phases of female reproductive milestones, such as menarche, pregnancy, and menopause [6, 7]. This association is likely linked to the increased frequency of migraine attacks during periods of relatively rapid fluctuations in sex hormone plasma levels, whereas lower attack frequencies are observed during intervals characterised by more stable hormonal conditions, such as the gradual rise occurring throughout pregnancy or the gradual decline during menopause. Additionally, menstrual cycle phases correlate with an increased incidence of migraine attacks during the perimenstrual phase [8, 9]. This is referred to as menstrual migraine, which often begins around the onset of menstruation and is estimated to affect 16–25% of women with migraine [9].

The high prevalence of migraine among females has prompted investigations into its genetic basis; however, despite three decades of research, no definitive explanation has emerged [10]. Scientists subsequently turned to genome-wide association studies (GWAS), which analyse millions of polymorphisms in large patient cohorts. In 2010, the first GWAS identified a single migraine susceptibility locus. A 2016 GWAS involving 59,674 migraineurs and 316,078 controls identified 38 distinct genomic loci associated with migraine [11]. A more recent GWAS reported 123 loci, although these were evenly distributed across all chromosomes and not specifically linked to the X chromosome [12]. Further GWAS, encompassing 102,084 migraine cases and 771,257 controls, revealed additional risk loci, including genes encoding calcitonin gene-related peptide (CGRP), a key target of two migraine-specific drug therapies [12]. Transcriptome-wide association studies (TWAS) have also been employed, comparing three TWAS methods to validate GWAS findings and identify novel risk gene loci [12, 13]. To date, nearly 200 loci have been associated with migraine in GWAS; however, each accounts for only a small fraction of genetic risk, and collectively they do not explain the full heritability of migraine [14].

As male–female differences are not observed in children but begin to appear at puberty [2], this suggests a hormonal rather than genetic basis for the sex disparity [15]. This interpretation is supported by the lack of robust evidence for sex differences in GWAS. Moreover, migraine attacks fluctuate throughout a woman’s lifespan, aligning with hormonal changes during the menstrual cycle, pregnancy, postpartum, and menopause [16].

Despite these observations, there are indications that genetic factors may contribute to the phenotypic sex differences in migraine. Vetvik and MacGregor proposed that commonly cited genetic explanations—such as the condition being autosomal dominant in women and autosomal recessive in men, or migraine being a direct consequence of an inherited variant—may be overly simplistic [4]. Current evidence emphasises that migraine is polygenic and influenced by multiple factors. Its genetic architecture appears highly complex, involving interactions among hundreds of common small-effect variants, rare variants affecting regulatory regions, and potentially “private” variants with moderate to large effect sizes [14].

At present, it is widely accepted that female sex hormones are involved in and closely intertwined with the pathophysiology of migraine, although the precise mechanisms remain elusive [17, 18]. Oestrogen, progestins, and androgens represent three major classes of endogenous sex steroids, with oestrogen, progesterone, and testosterone being the most well-known hormones in each respective class. Ovarian steroids, including oestrogen and progesterone, exert profound influences on the central nervous system (CNS). These sex hormones can cross the blood–brain barrier (BBB) passively due to their lipophilicity or be synthesised locally within the CNS, where they exert wide-ranging and complex effects across multiple brain regions, including those involved in pain processing, sensory and autonomic functions, and affective regulation [19].

The high prevalence of migraine in women has prompted extensive efforts to understand migraine subtypes, particularly in relation to the menstrual cycle. This has led to the proposed classification of subtypes such as “pure menstrual migraine”, “menstrual-related migraine”, and “non-menstrual migraine” [20]. It is hypothesised that the well-documented rapid decline in circulating oestrogen levels prior to menstruation is not a direct trigger of migraine but rather reflects an endogenous neuroendocrine characteristic in females [9, 21]. Furthermore, migraine is most prevalent between the ages of 15 and 49 years, significantly impacting a woman’s quality of life, as well as her family, employment, and social responsibilities [4, 22]. According to the Global Burden of Disease study, it ranks as the most disabling condition within this age group [3]. A large longitudinal study demonstrated that women experience more frequent attacks and are more likely to be disabled by them compared with men [23]. Headache and associated symptoms occur more often in women [24], and the duration of attacks—whether with or without aura—is longer in women than in men [25]. In menstrual-related migraine, contraceptive pills are commonly used in clinical management; however, responses vary considerably, and success rates show substantial variability [4, 5, 9].

## Involvement of sex hormones

As early as 1972, Somerville proposed the *oestrogen withdrawal hypothesis*, suggesting that a decline in oestrogen levels during the late luteal phase triggers migraine attacks [26–29]. The role of oestrogen withdrawal at the onset of the menstrual cycle has since been widely accepted as a key trigger of migraine attacks in women [30]. However, there is still no molecular explanation for the mechanism underlying this effect of oestrogen, nor any specific treatment targeting the molecular events responsible for menstrual-related migraine. Clinicians typically rely on standard care for these patients [9]. Since both oestrogen and progesterone show marked reductions at the onset of menstruation, they are often discussed as the main culprits. Progesterone has been described as modulating the effect of oestrogen in migraine, with declining levels accompanying the reduction in oestrogen just prior to menstruation [31–34]. Additionally, a recent study on progesterone provides further insight into the expression and co-localisation of these sex hormones within the trigeminovascular system (TGVS) and the CNS [6]. Progesterone and its receptor, progesterone receptor-A (PR-A), are present in the trigeminal ganglion (TG). While progesterone is located predominantly in cell membranes and Aδ-fibres, PR-A is found in neuronal cytoplasm and nuclei, as well as in satellite glial cells [35].

Research over the past decade has also highlighted the hypothalamus as a crucial region in migraine initiation [36, 37], implicating hypothalamic hormones in this process. From a pathophysiological and neurohormonal perspective, this part of the brain contains two clusters of large cell bodies—the paraventricular (PVN) and supraoptic (SON) nuclei—which produce two neuropeptides: oxytocin and its molecular relative, arginine vasopressin (AVP). Both are released into the posterior pituitary and subsequently into the circulation [38, 39]. In addition, nerve fibres from the PVN and SON project to the brainstem trigeminal nucleus caudalis (TNC), which serves as the central hub for peripheral input from the TGVS [38, 40].

## The trigeminovascular system and head pain

The TGVS has been a focal point in migraine research for decades. Recently, it was demonstrated that this component of the pain system is extensively populated by oestrogen receptors [41]. The TGVS comprises the TNC, the TG, the trigeminal nerve, and its peripheral branches [42]. The peripheral structural organisation, known as V1–V3, provides sensory innervation to different regions of the head and cranium.

Neurons within the TG are generally categorised into two main types: small to medium-sized neurons that primarily produce CGRP, which is stored in the cell bodies and transported via axoplasmic flow in C-fibres; and medium to large-sized neurons (> 45 μm in diameter) that contain CGRP receptors and are present in Aδ-fibres [42]. Several other neuropeptides and small molecules are also found in the TGVS, albeit at lower levels [43]. Many primary headache symptoms are associated with activity within the TGVS, and the largest group of headaches—migraines—can be successfully treated with drugs that modulate the activity of the CGRP family of peptides [44]. The sensory neuropeptide CGRP is consistently shown to be released from the TGVS following acute migraine attacks [44]. However, CGRP release from the TGVS during a migraine attack accounts for only about one-fifth of the total CGRP released during such episodes [45], complicating the use of plasma CGRP as a biomarker for migraine.

To date, no significant sex differences have been demonstrated in CGRP release during spontaneous migraine attacks or in experimental models [46, 47], nor in therapeutic responses to CGRP-targeted drugs such as monoclonal antibodies (mAbs) and gepants [48, 49]. Clinical studies have not shown differences in efficacy between male and female migraine patients treated with gepants or mAbs [17, 45]. Similarly, GWAS studies have not revealed sex-specific differences [10]. Phase III and phase IV clinical trials of mAbs and gepants have included both sexes, yet no significant differences in responsiveness have been observed between male and female patients for mAbs targeting CGRP signalling (erenumab, fremanezumab, galcanezumab, eptinezumab) or the three available gepants (ubrogepant, rimegepant, atogepant), all approved for migraine treatment [50].

Despite the high global prevalence of migraine, particularly among females, few studies have explored the specifics of sex differences and the role of hormones in migraine models. Current knowledge regarding sex hormone modulation of migraine headache largely depends on a limited number of animal studies [51], with most information derived from subgroup analyses of clinical trial data.

## Sex hormone fluctuations as a trigger of migraine

The focus of this review revolves around three essential aspects of migraine pathophysiology: (i) the TGVS is closely linked to pain and sensitisation in migraine; (ii) the ability of hormones to cross the BBB may influence the TGVS, which itself lacks BBB protection; and (iii) the local production of sex hormones within the CNS, enabling modulation of migraine pain through both central and peripheral signalling mechanisms.

Migraine follows a characteristic temporal pattern throughout a woman’s life, aligning with sex hormone fluctuations during key reproductive milestones. Puberty marks a critical period when migraine and headache begin to affect women more significantly. In contrast, the trajectory of migraine across a man’s lifespan appears relatively stable. This may correlate with the gradual decline in levels of autonomic neuropeptides (e.g., neuropeptide Y, vasoactive intestinal peptide) and sensory neuropeptides (e.g., CGRP, substance P) from birth to old age in both sexes [52]. Older patients often report that migraine persists into advanced age, but the headache becomes milder or even disappears [53].

For perimenstrual migraine, it is hypothesised that low estradiol levels (< 50 pg/mL) are associated with reduced migraine prevalence in menopausal women [54, 55]. Women with migraine generally experience improvement after spontaneous menopause; in contrast, surgical menopause often results in worsening symptoms [56]. The reason why fluctuations in circulating oestrogen promote menstrual-related migraine remains unclear. It has long been proposed that oestrogen withdrawal may lead to sensitisation of the TGVS, modulation of neurotransmitters, increased synthesis of neuropeptides, and altered microglial reactivity [9, 57, 58]. In the CNS, low serum levels of oestrogen, progesterone, and magnesium—combined with elevated uterine prostaglandin concentrations—are thought to exert pronociceptive effects by modulating glutamatergic signalling and promoting increased expression of brain-derived neurotrophic factor (BDNF) and nerve growth factor (NGF) [58]. Oestrogen serum levels can also influence local serotonin (5-HT) concentrations through downregulation of its rate-limiting synthetic enzyme, tryptophan hydroxylase (TPH) [59]. Monoamine oxidase A (MAO-A) is the primary catalytic enzyme responsible for 5-HT degradation. Interestingly, MAO-A activity has been shown to be significantly greater during the post-ovulatory phase compared with the pre-ovulatory phase of the menstrual cycle [60], suggesting an inverse relationship between circulating oestrogen levels and MAO-A activity. If a similar mechanism operates within the TGVS, an oestrogen-mediated reduction in 5-HT levels could indirectly increase local concentrations of released CGRP and, at the same time, remove the inhibitory “brake” on the cAMP signalling pathway—plausibly leading to further sensitisation of the trigeminal system.

Recently, we examined whether oestrogen or progesterone exert a direct effect on stimulated CGRP release from the TGVS [6, 41]. Interestingly, neither hormone had a direct effect on CGRP release, suggesting an indirect or modulatory role, possibly via nuclear receptors [41]. Oestrogen acted as a modest vasodilator, while progesterone exhibited weak contractile properties [61]. Since both hormones exhibit opposing and relatively weak vascular effects, they are unlikely to be directly responsible for triggering menstruation-related migraine attacks. The role of progesterone is less clear since it is a vasoconstrictor and did not modify the CGRP release, still it showed a drop in circulating levels at the start of menstruation.

The predominance of migraine in females coincides with menarche, marked by a doubling of migraine attacks during the perimenstrual period compared with other phases of the menstrual cycle. Menstruation is a well-recognised trigger, with 18–25% of women with migraine experiencing attacks during this time. Comparisons between women with and without migraine reveal that those with migraine exhibit a more rapid decline in oestrogen during the late luteal phase [9]. Consequently, the timing and rate of oestrogen withdrawal have been proposed as markers of vulnerability to migraine in women.

The oestrogen withdrawal hypothesis suggests that a decrease in oestrogen promotes nociceptive signalling within the TGVS, thereby triggering migraine attacks [8]. Oestrogen rapidly regulates membrane hyperexcitability in the TG via oestrogen receptor alpha (ERα) and exerts anti-nociceptive effects [62, 63]. According to this theory, a drop in oestrogen reduces anti-nociception at the level of the TG. However, the precise mechanism remains unclear: does this occur via nuclear ERα, or through cytoplasmic ERβ and GPER (G protein-coupled oestrogen receptor 1), all of which are located in the TGVS [41]. Migraine demonstrates a complex relationship with pregnancy. Generally, women with migraine experience symptom improvement during the third trimester. However, in up to 1–10% of pregnant women, migraine may begin or worsen, affecting approximately 8% during the first trimester [64]. Recently, Holm et al. demonstrated that during the menstrual cycle in mice, or following exogenous oestrogen administration, the expression of genes encoding CGRP and receptor activity-modifying protein 1 (RAMP1)—a key component of the canonical CGRP receptor—was altered [65]. This finding may provide an important clue to understanding how hormones regulate pain in menstrual-related migraine.

Oestrogen hormone replacement therapy, or menopause replacement therapy (MRT), typically involves continuous dosing of oestrogen alone or in combination with progestin. A major limitation of these therapies is that they do not relieve migraine in all women; in some cases, headaches may even worsen. Furthermore, MRT carries an increased risk of heart disease, stroke, thromboembolism, and breast cancer [66, 67].

Fluctuations in oestrogen and progesterone are believed to influence migraine pathogenesis [68]. Notably, many women report the onset of migraine attacks just prior to menstruation [5]. One theory suggests that the rapid reduction in circulating oestrogen not only triggers menstruation but also initiates menstruation-related migraine attacks [9]. Recently, it was shown that oxytocin levels also decline sharply in parallel with oestrogen, potentially contributing to headache initiation [8]. We propose an alternative perspective: this reduction in hormone levels effectively “*letting the brake off*” on TGVS activity, which plays a central role in migraine.

In the following sections, we will explore how alterations in sex hormone levels may trigger mechanisms associated with migraine onset. Understanding the molecular basis of hormonal influences on the TGVS will empower women to better manage their migraine symptoms.

## How can sex hormones and their fluctuations modify pain?

While migraine also affects men, its underlying cause cannot be attributed solely to dynamic changes in female hormones. We have demonstrated that receptors for both sex hormones are present in males, albeit at somewhat lower levels [6, 41]. These hormones are also produced at sites outside the female reproductive system; one notable source is the vascular endothelium. However, it is plausible that oestrogen and progesterone, with their characteristic fluctuations during various stages of a woman’s life and menstrual cycle, modulate specific receptors located both in the CNS and the TGVS. Nevertheless, there is little doubt about their importance in migraine among women. Additionally, lifestyle and environmental factors may influence migraine onset, but the greatest risk factor remains female sex.

Due to their ability to cross the BBB, circulating sex hormones can directly and indirectly influence the synthesis, neuronal firing, and secretion of hypothalamic hormones such as oxytocin and AVP [69, 70]. Notably, neurons in the PVN and SON express ERα, ERβ, PR, and androgen receptors. Activation of ERβ within the PVN has been shown to increase the transcription and synthesis of intracellular oxytocin [71]. In addition, low blood pressure, detected via baroreceptors, is known to stimulate hypothalamic AVP release. The neuroendocrine circuitry is highly complex, and further research is required to fully elucidate its contribution to headache pathogenesis.

The high prevalence of migraine is linked to dynamic changes in circulating ovarian steroids [51, 72], as well as hypothalamic oxytocin and AVP, from menarche to menopause. These hormones critically regulate not only numerous physiological processes but also migraine. Moreover, migraine and oestrogen withdrawal are associated, with rising oestrogen levels providing a protective effect against cardiovascular disease [16]. During menopause, when hormone levels are low, migraine incidence decreases. In the second and third trimesters of pregnancy, oestrogen and progesterone levels are high, whereas postpartum migraine often recurs [27, 73]. There also appears to be a relationship with circulating CGRP, which is notably elevated during pregnancy and influenced by oral contraceptives containing high levels of oestrogen [74]. Precisely how these hormones regulate CGRP and its receptor, or other neuromodulators, requires further investigation.

In migraine without aura, attacks are reduced during pregnancy [75], when ovarian hormone levels in serum are stable and high. Conversely, in migraine with aura, new attacks often occur during pregnancy [76]. The use of oral contraceptives may exacerbate migraine with aura [76], and this may also be true for migraine without aura. Several neurotransmitter systems and pain-processing networks are thought to play important roles in migraine pathogenesis and can be modulated by oestrogen and progesterone [54, 55]. An interesting study by Pardutz et al. [77] investigated nitroglycerine-mediated CGRP and 5-HT expression in the superficial laminae of the TNC in oestrogen-treated and vehicle-treated oophorectomized rats. Immunohistochemical analysis revealed that CGRP expression in the vehicle-treated group was significantly lower compared with the oestrogen-treated group. The results further showed that the area occupied by 5-HT-immunoreactive neurons was significantly greater in the oestrogen-treated animals than in the vehicle-treated controls, suggesting that oestrogen treatment can modulate basal 5-HT expression in the rat TNC.

It appears that the TGVS plays a key role in explaining many migraine symptoms and that variations in hypothalamic and sex hormones may act as modulators of signalling. This notion is supported by Ibrahim [78], who observed that capsaicin-induced responses varied in females depending on the phase of the menstrual cycle.

## Localisation of oestrogen and its receptors

Oestrogen, a pivotal hormone, is primarily synthesised in the female reproductive tract but also in other parts of the body, including the vascular endothelium, and plays a crucial role in numerous physiological processes [79]. This aligns with measurable levels of oestrogen also being present in men. The hormone exerts effects across many regions of the body, including bone formation, the CNS, and the cardiovascular system. Due to its lipid solubility, steroid hormones such as oestrogen can cross the BBB and modulate brain function, although this area of research remains underdeveloped. Studies have demonstrated three types of oestrogen receptors (ERα, ERβ, and GPER) [80], which are expressed in migraine-related anatomical structures such as the TGVS and the CNS [41].

It is evident that oestrogen influences the occurrence of attacks in women who are biologically predisposed to migraine. Current hypotheses, supported by GWAS data, suggest no clear genetic difference between males and females in migraine susceptibility [10]. Perimenstrual administration of oestrogen to women prone to menstrual-related migraine has been shown to significantly reduce attack frequency [81, 82]. Similarly, oral contraceptives can be preventive; however, acute oestrogen treatment does not abort an ongoing migraine attack [9]. Discontinuation of contraceptive use correlates with an increase in attacks, consistent with the withdrawal theory [28]. Current evidence therefore points towards menstrual-related migraine attacks being associated with a decrease in circulating hormone levels, particularly oestrogen [8, 29]. Yet the key question remains: *why does a decrease in oestrogen appear to trigger an attack?*

**Fig. 1:**
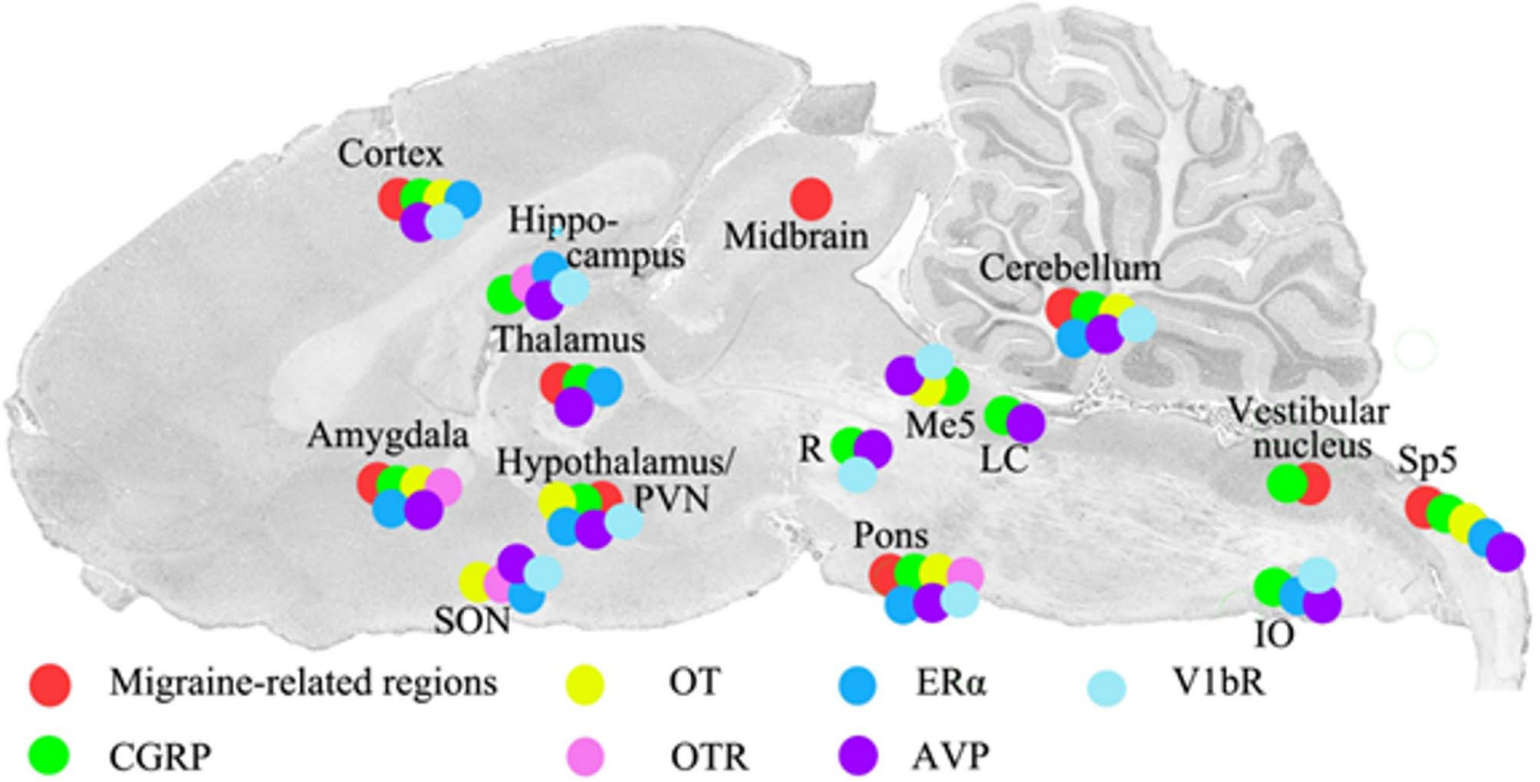
Sagittal cryo-sections of the rat brain (schematic reworked from [38]). The coloured dots on the sections represent the immunohistochemical mapping of hormones and their receptors in the volume spanning 0.5 to 1.5 mm from the midline of the brain. Our group has earlier examined the distribution of hormones and their receptors [38–40]. The present figure shows the migraine related regions, CGRP, oxytocin, oestrogen, AVP and related receptors. OT: Oxytocin, OTR: Oxytocin receptor, ERα: Estrogen receptor alpha, AVP: Vasopressin, V1bR: Vasopressin receptor 1b

The distribution of oestrogen receptors in the CNS [41] revealed that ERα expression is mainly observed in the nuclei of neurons and glial cells in certain areas (e.g., corpus callosum) (Fig. 1). Moreover, processes from hippocampal pyramidal cells express ERα. ERβ is primarily observed in the hippocampus and cerebellum [41]. Notably, ERβ is absent from brain regions known to be involved in migraine pathophysiology, such as the cortex, thalamus, amygdala, hypothalamus, pons, and TNC [41].

In contrast, GPER is mainly found in the pons, the cerebellar molecular layer, and importantly in the spinal trigeminal tract (TNC/Sp5), a sensory tract and key area linking the CNS with the periphery via the TGVS [41]. This region relays sensory modalities including temperature, deep or crude touch, and pain from the ipsilateral portion of the face and head. The spinal trigeminal tract constitutes an essential part of the pain pathways activated during migraine attacks.

ERα is a well-known nuclear receptor, but it has also been observed in satellite glial cells and in Aδ fibres within the rat TG [83]. ERβ has been observed in the cytoplasm of TG neurons, exhibiting a staining pattern resembling that of the Golgi apparatus. Furthermore, immunohistochemistry revealed that ERβ and CGRP antibodies co-localised, suggesting expression within the same organelle (the Golgi apparatus).

GPER and CGRP are co-expressed in most CGRP-positive cells [41]. Recently developed genetic tools and chemical ligands have greatly facilitated research aimed at determining the physiological roles of GPER in different tissues. However, evidence that GPER plays a significant role in mediating endogenous oestrogen action in vivo remains lacking [84]. Importantly, oestrogen receptors are expressed in the same neurons that express both CGRP and CGRP receptors, further supporting the hypothesis that sex hormones may modulate the CGRP system, which plays a pivotal role in migraine pathophysiology [44].

While the trigeminal system contains all three types of oestrogen receptors in both males and females, a significant sex difference in expression is observed only for ERβ. Based on this hypothesis, Holm and colleagues investigated how sex hormones regulate CGRP-related genes in the TG, focusing on RAMP1, using both wild-type (WT) and *Ramp1* knockout (KO) mice [65]. The expression of *Ramp1* varied across the oestrous cycle, peaking in proestrus and declining in diestrus, inversely correlating with the gene encoding CGRP (*Calca*). The gene encoding the canonical CGRP receptor, CLR (*Calcrl*), remained unchanged, while *Ramp1* expression correlated significantly with *Esr2* (encoding ERβ), suggesting oestrogen receptor-mediated regulation. Oestrogen treatment upregulated *Ramp1* in both sexes; *Calca* was downregulated in females but upregulated in males. Progesterone had more modest effects, primarily altering *Ramp3* expression. In *Ramp1* KO mice, the cyclical variation of *Calca*, *Ramp2*, and *Ramp3* seen in WT mice was absent, and basal *Calca* expression was elevated in males, indicating that RAMP1 is essential for hormonal regulation of the CGRP system. Additionally, these findings support a role for oestrogen-driven epigenetic mechanisms, such as DNA methylation, in the long-term regulation of *Ramp1* [65]. It is reasonable to conclude that sex hormones modulate the CGRP system and/or act at the mRNA level to exert longer-lasting effects via transcriptional regulation of neuronal function.

## Progesterone and its receptors

Progesterone, classified as a progestin, plays crucial roles in the menstrual cycle, pregnancy, and embryogenesis [85, 86]. Like oestrogen, it is synthesised in the ovaries, but also in the adrenal glands and placenta [87]. Progesterone is transported through the bloodstream to target cells and stored in adipose tissue. Its levels are much higher than those of oestrogen and exhibit even greater dynamic fluctuations throughout a woman’s lifespan, including a sharp decline in circulating levels at the onset of menstruation [88]. Peak levels occur during the maturation and release of the egg.

**Fig. 2:**
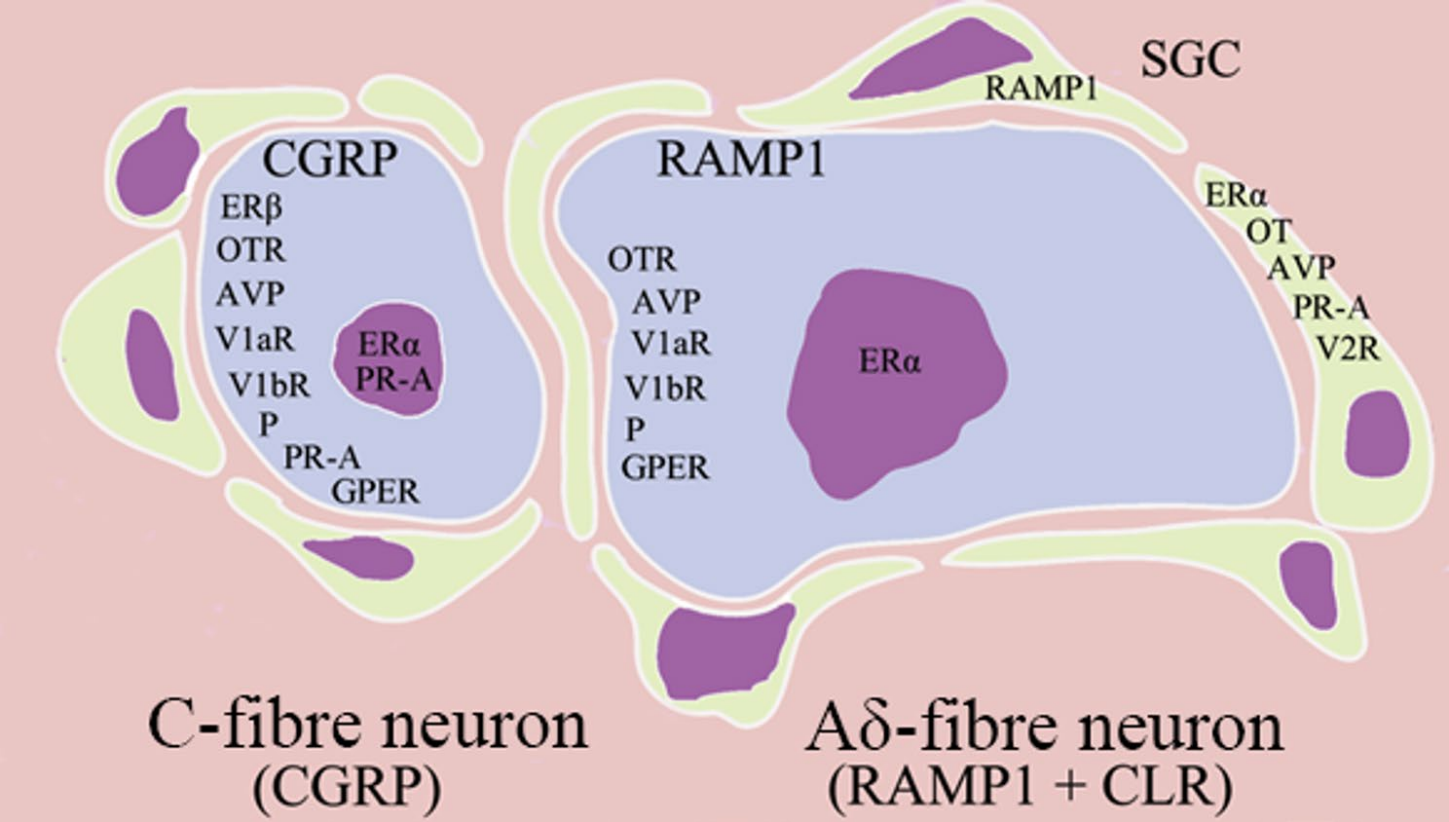
TG distribution of CGRP, RAMP1 (receptor activity-modifying protein 1), oestrogen receptors (ERα, ERβ, GPER), AVP, vasopressin receptors (V1aR, V1bR, V2R), oxytocin (OT), oxytocin receptor (OTR), progesterone (P), progesterone receptor (PR-A) in C- and Aδ-fibre neurons and satellite glial cells (SGC). TG mainly contains two types of neurons; one small to medium sized that produce CGRP stored in the cell bodies and in the C-fibres, while the medium sized to large neurons contain CGRP receptors (RAMP1/CLR) which are present in Aδ-fibres. The sketch illustrates the two different types of neurons, and the satellite glial cells (SGC) enveloping the neurons. Within each cell type, expression of hormones and receptors is mapped. The SGCs contain yellow cytoplasm and violet nucleus, and the neurons light blue cytoplasm and violet nucleus

Recent studies have revealed co-expression of progesterone and its receptor, PR-A, with CGRP and its receptor components—calcitonin receptor-like receptor (CLR) and RAMP1—in different parts of the TGVS [6]. The TG displays progesterone expression in or near the cell membranes of large to medium-sized neurons and in thin myelinated nerve fibres (Aδ fibres) (Fig. 2A). Since progesterone mRNA levels were very low, it is proposed that progesterone is not synthesised locally in the TG but is present due to binding of circulating progesterone to cell membranes [35]. The progesterone receptor PR-A was observed in both the nucleus and cytoplasm of satellite glial cells (SGCs) [6, 89].

Progesterone exhibits diverse functions in various parts of the body, including anti-inflammatory and anti-vasoconstrictive effects, and may have a neuroprotective role [90, 91]. It is closely linked to the trigeminal system, with PR-A expression in Aδ fibres and CGRP-containing neurons, suggesting a possible role in headache mechanisms.

Although progesterone fluctuates during different reproductive stages, changes in plasma progesterone levels are not directly correlated with migraine attacks [6]. Furthermore, experimental studies of CGRP release from different parts of the TGVS revealed no acute direct influence of progesterone on either basal or stimulated CGRP release [6]. Progesterone metabolites have been reported to modulate several neurotransmitters, such as GABA and glutamate, in the CNS, and participate in neuron–glia interactions [92]. Notably, allopregnanolone has been shown to inhibit substance P–induced plasma protein extravasation in the rat dura mater in a dose-dependent manner [93]. This finding suggests that progesterone, and its metabolites, may influence meningeal inflammation, which could be relevant to the pathophysiology of migraine. Additionally, progesterone can modify the activity of mitogen-activated protein kinases (MAPKs), such as MEK–ERK1/2 [94]. However, limited knowledge exists regarding the expression and functional role of progesterone receptors in migraine-related regions of the CNS and within the TGVS.

## Testosterone and its receptor

The male sex hormone testosterone (*17β-hydroxyandrost-4-en-3-one*) is the most important of the androgens. It is a steroid hormone that can be synthesised from progesterone and may act independently or as an intermediate in the formation of oestrogen. Testosterone is primarily produced in the testes and, to a lesser extent, in the ovaries, with minor contributions from other tissues. Its levels are approximately ten times higher in men than in women. In women, testosterone promotes growth, reproduction, and general health; however, aromatisation of testosterone into oestrogen becomes the main source of oestrogen production after menopause [95]. During the menstrual cycle, serum testosterone levels are generally low in the early follicular and luteal phases but exhibit a surge around ovulation [96]. The effects of testosterone are mediated via androgen receptors, which are involved in cellular transcription.

Little research has explored the role of testosterone in headache disorders; however, it may contribute to sex differences. This hormone alone cannot explain the fluctuating susceptibility observed in women. Intriguingly, male-to-female transgender individuals with migraine exhibit changes in attack frequency when treated with oestrogen, underscoring the influence of oestrogen in migraine pathophysiology [97].

Testosterone has been considered a potential therapeutic target due to its partial antinociceptive effect [98–101]. In animal studies, gonadectomy or blockade of testosterone receptors appears to increase sensitivity to nociceptive stimuli [102–105]. A few human studies support an analgesic effect of testosterone, as higher testosterone levels are associated with lower experimental pain sensitivity [106]. Research on the relationship between testosterone and migraine remains limited. Available evidence suggests that testosterone levels are lower in adults with migraine compared to those without and correlate with migraine severity. Several migraine-related peptides exert strong vasomotor effects [107], whereas testosterone demonstrates only a weak dilatory effect on human cranial arteries [61].

## Hormonal regulation of the TGVS

Today, ample information exists on the expression of oestrogen and progesterone in the CNS as well as in the PNS (peripheral nervous system), and on oestrogen expression in vessel walls. The pain system for the head region is primarily associated with the TG, which connects peripherally to cranial structures and centrally to the brainstem and pain-processing centres. However, limited data exist on how pain-related molecules and their receptors within the TGVS influence expression and function in relation to head pain.

A recent study by Holm et al. demonstrated a correlation between oestrogen receptor expression and the expression of CGRP and RAMP1 genes [65]. Their close association with CGRP and its receptor suggests potential effects on baseline CGRP levels, as well as on pituitary adenylate cyclase-activating polypeptide (PACAP) release, which was higher in pro-oestrous compared with oestrous phases [108]. In recent years, attention has increasingly focused on the role of PACAP38 in headache disorders [103], particularly in relation to sex hormones [109], particularly in relation to sex hormones [72]. Stimulated neuropeptide release induced by high-potassium depolarisation did not reveal any sex differences in experimental models. Vasomotor studies of sex hormones on human intracranial arteries demonstrated dilatory responses in both rats and humans, but no sex-related differences were observed [61]. In contrast, hypothalamic hormones such as oxytocin and AVP are strong vasoconstrictors, whereas ovarian hormones only modestly relaxed human intracranial arteries [8, 40, 41, 110].

These findings suggest that the pro-migraine effect of oestrogen is not primarily vascular per se but may instead involve a modulatory role on trigeminal neurons via transcriptional modification of the CGRP system [41, 65]. Progesterone, on the other hand, did not alter basal or stimulated CGRP release in the TG or dura mater in either males or females [35], nor did it affect transcription of the CGRP system [65]. Additionally, progesterone enhanced the dilatory response to capsaicin in male basilar arteries [6]. To date, only minor sex-related differences have been observed for progesterone in relation to the TGVS.

## Hypothalamus, a key player in migraine

A typical migraine attack begins with premonitory symptoms that may appear 1–2 days before the headache phase, followed by a throbbing unilateral headache that can last up to 72 h [1, 111, 112]. The underlying pathophysiology remains a subject of debate, and various hypotheses have emerged to explain the complexity of migraine biology. May and colleagues demonstrated using fMRI that signs of hypothalamic activation occur as early as 1–2 days before the headache phase [36, 37]. Interestingly, the posterior hypothalamus appears to be important for acute pain, while the anterior region may be involved in attack generation and migraine chronification [113]. How this activity initiates a migraine attack is still unclear, but during the headache phase, cortical, thalamic, and brainstem activation is observed. These findings challenge the early vascular theory proposed by Graham and Wolff, which suggested that migraine attacks originate in cranial vasculature [114], a concept supported by experimental intravenous infusion studies of vasodilators in humans [115, 116]. GWAS studies have also indicated a role for vascular genes [11, 117], as well as mechanisms within the TGVS involving trigeminal sensory nerves, mast cells, inflammation, and sensitisation [118].

**Fig. 3:**
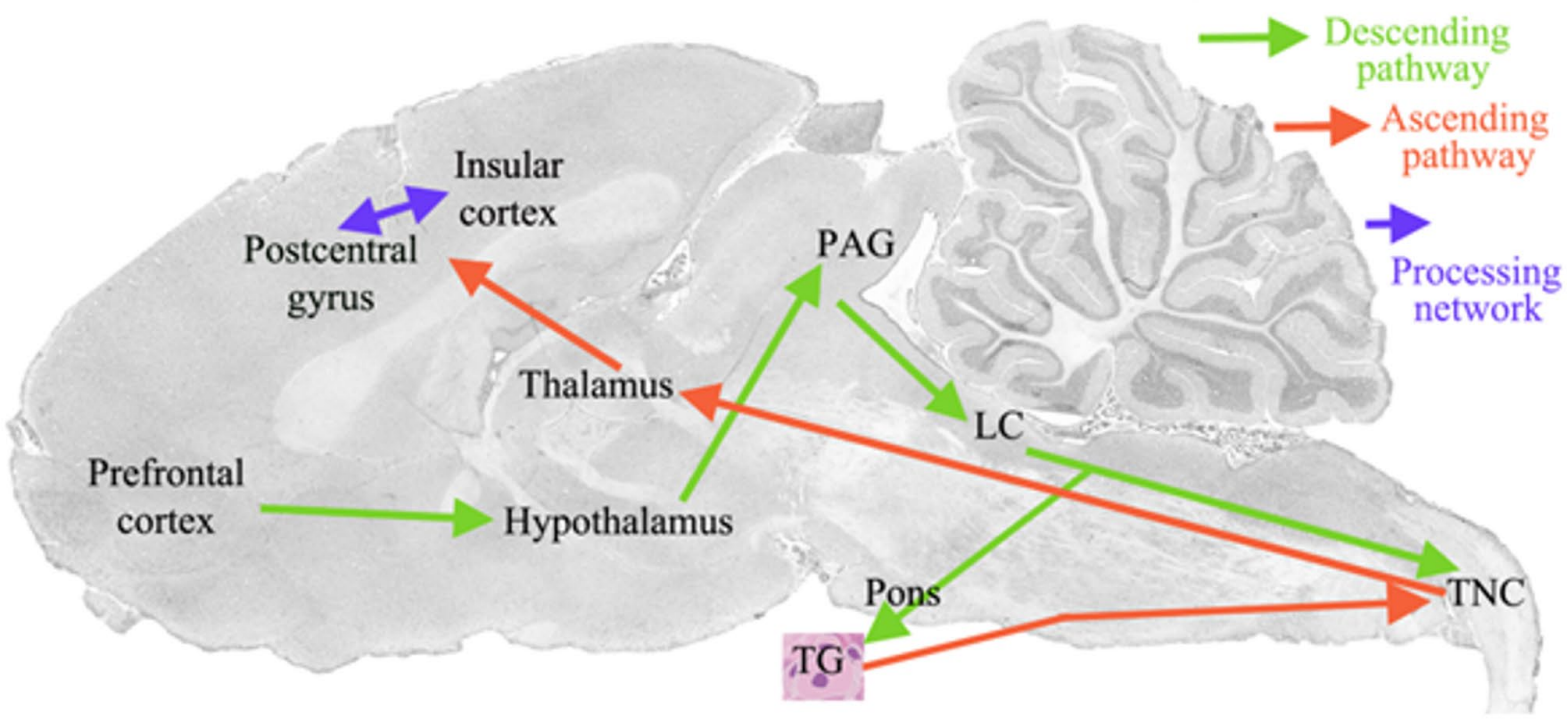
Overview of migraine related pathways. The schematic overview demonstrates the ascending projections from TG to TNC (C1-C3 according to tracing studies by Liu, Broman and Edvinsson [163]), thalamus and cortex The descending projections start at prefrontal cortex to hypothalamus, PAG, LC and to TNC

The CNS origin of migraine has gained further support from functional neuroimaging studies by May, Goadsby, and Burstein, which collectively revealed that migraine attacks are initiated in the hypothalamus, with connections to CNS regions such as the thalamus and brainstem, and involve typical neurological features [119, 120]. Based on current understanding of premonitory symptoms, the hypothalamus is considered the most likely initiator of migraine attacks [121]. Cluster headache attacks often occur at night and are linked to reduced circulating melatonin levels. Melatonin acts on receptors in numerous brain regions involved in pain processing and on the master circadian clock in the hypothalamic suprachiasmatic nucleus (SCN), which expresses melatonin receptors [122]. This provides further evidence for hypothalamic involvement in initiating primary headache attacks. Hypothalamic activity is also coupled to the brainstem, particularly the TNC, during the ictal stage. This aligns with early PET studies by Weiller et al. [120] and was confirmed by Bahra and Goadsby [120, 123], which verified activity in brainstem regions during the headache phase of migraine. In both males and females, several brain regions are implicated in migraine, including the cortex, thalamus, hypothalamus, amygdala, midbrain, pons, cerebellum, vestibular nucleus, and the TNC region (Fig. 3) [124]. Another proposed migraine initiator is cortical spreading depression (CSD) [125], which has been shown to activate the TGVS and produce aura symptoms [111, 126]. Recent evidence suggests that aura may be an epiphenomenon rather than the cause of migraine [127]. Supporting this view, Arne May demonstrated that aura can occur several days before the actual migraine attack [128]. Collectively, dynamic imaging provides strong evidence for the association between the hypothalamus, the TGVS, and migraine symptoms (Fig. 3).

Activation of the TNC may lead to downstream activation, potentiation, or modulation of TGVS function, either through direct activation or altered sensory signalling. This is plausible since the TNC lies beyond the reach of many effective anti-migraine medications (such as mAbs) [129–131]. A mismatch in TGVS communication or tuning may result in sensitisation and potentially trigger CGRP release, shifting the migraine attack from a central to a peripheral process. While NSAIDs and ditans can cross the BBB to some extent, triptans show limited penetration (approximately 3%). Older medications such as antiepileptics, antidepressants, and some β-adrenoceptor blockers exhibit significant BBB permeability.

Recent advances in understanding migraine mechanisms, particularly at the molecular level, offer new insights for developing novel treatments, such as those targeting nodes of Ranvier [132]. Current drugs include small-molecule gepants and mAbs, which have limited BBB penetration at pharmacologically effective concentrations [131]. Consequently, therapeutic strategies primarily target the trigeminal system located outside the BBB, which is accessible to circulating drugs [133]. Circulating hormones such as oestrogen, progesterone, and oxytocin act at these sites and may exert modulatory effects on the TGVS. These hormones are lipophilic and small molecules, enabling passage across the BBB [134]. Nevertheless, future research must explore strategies beyond the current formulary of small, lipophilic molecules to deliver therapeutics effectively to sites within the CNS.

## Oxytocin and its receptor

The hypothalamic peptide oxytocin has been recognised for its anti-migraine effects for over two decades [135], a finding later confirmed in two independent studies [136, 137]. Oxytocin and AVP are expressed in clusters of large neurons within the SON and PVN of the hypothalamus [39]. These neurons project fibres to the posterior pituitary gland, where oxytocin and AVP are released into the bloodstream. Both hormones circulate at significant levels and exhibit high evolutionary stability in their molecular structure [138]. Oxytocin release into the bloodstream regulates endocrine events associated with reproduction in both sexes [139]. Additionally, oxytocin is released from the cell bodies and dendrites of these neurons [140].

Oxytocin-containing fibres project widely throughout the brain, with receptors located in regions relevant to migraine pathophysiology, such as the brainstem and TNC [38, 40]. Notably, recent studies have demonstrated substantial expression of oxytocin receptors in the TGVS, co-localising with CGRP and the CGRP receptor components CLR/RAMP1 [40]. These findings suggest that oxytocin may play a central role in migraine pathophysiology and could be pivotal in advancing our understanding of hormonal influences on migraine [38–40]. Experimental studies of the TGVS have shown oxytocin receptor protein and mRNA in the TG, localised in both C-fibre and Aδ-fibre neurons [40]. The TNC also expresses oxytocin receptor protein and mRNA, supporting a functional link with PVN and SON projections.

CGRP release experiments from isolated TG and dura mater were not altered by oxytocin administration [40]. Studies of oxytocin and AVP revealed strong vasoconstriction of isolated rat and human cerebral and meningeal arteries, although such high concentrations are not observed in patients [61]. Pharmacological analysis using AVP V1A receptor blockers demonstrated that the contractile response to oxytocin occurs via the AVP V1A receptor rather than a vascular oxytocin receptor [40]. The precise mechanisms and sites of oxytocin involvement in migraine pathophysiology require further clarification [141].

Recent work confirms that the TGVS contains oxytocin receptors [39, 40]. These receptors are present in numerous small to medium-sized neurons and thick axons characteristic of Aδ sensory fibres, which also contain CGRP receptors [40]. Only a small subset of neurons co-express both oxytocin receptor and CGRP (C-fibres). Oxytocin itself is expressed only to a minor extent in satellite glial cells of the TG. It has been proposed that oxytocin may block CGRP release from trigeminal dural afferents, suggesting a potential therapeutic benefit in migraine and other primary headache disorders [142]. However, a recent study could not confirm that oxytocin inhibits CGRP release from the dura mater in rats [40]. Only a small fraction of oxytocin crosses the BBB and likely acts on receptors within the TGVS to modulate nociceptive mechanisms [39, 40].

“*The oxytocin theory of migraine”* is a complementary hypothesis suggesting that a reduction in oxytocin may influence nociceptive processing in regions such as the brainstem and the TGVS. Recent studies have highlighted the potential involvement of oxytocin in migraine pathophysiology [8], particularly in relation to menstrual-related migraine, where its inhibitory effects on pain and nociception are significant [57, 143–146]. An analgesic effect of oxytocin–oxytocin receptor binding has been proposed to occur via a potassium channel/nitric oxide/KATP pathway [57, 144]. The effect of oxytocin on migraine was first reported in a case study where intravenous oxytocin provided analgesia and migraine relief [135]. Furthermore, double-blind, placebo-controlled clinical trials have shown that intranasal oxytocin sprays are effective in treating migraine pain in adults of both sexes [136, 137] and in reducing experimentally induced pain in men [147]. Importantly, intranasal oxytocin in humans has not been associated with major side effects [148]. Although the oxytocin theory of migraine is still in its infancy, accumulating evidence suggests a link between oxytocin and migraine pathophysiology. Oxytocin’s involvement in headache sensitivity, stress modulation, vasodilation, and 5-HT pathways indicate a potential role in migraine. Studies on pregnancy have shown that increased circulating oxytocin levels can reduce migraine severity [57, 149, 150]. Moreover, intranasal oxytocin has been shown to relieve headaches in a dose-dependent manner [137, 151].

CNS studies have demonstrated that oxytocin and its receptor are present in several brain regions associated with migraine [39]. As expected, oxytocin is extensively localised in the cell bodies of neurons within the hypothalamic SON and PVN. The most prominent oxytocin receptor expression occurs in the hippocampus, pons, and substantia nigra. In some regions (e.g., the amygdala and hypothalamus), both oxytocin and its receptor are co-expressed, whereas mismatches between peptide and receptor distribution are observed in the cerebral and cerebellar cortex (oxytocin expression) and hippocampus (oxytocin receptor expression). Comparisons of oxytocin/oxytocin receptor distribution with CGRP mapping reveal overlap in regions previously identified as “migraine generators” [120], suggesting that central oxytocin pathways may contribute to hypothalamic involvement in migraine attacks.

Oxytocin’s therapeutic effects in migraine are complex, as the peptide is widely distributed throughout the nervous system, including primary sensory neurons, the spinal cord, and multiple brain regions involved in pain processing and modulation [136, 152, 153]. A recent theory proposes that menstrual-related migraine is associated with a decline in both oestrogen and oxytocin immediately before and during early menstruation [8]. Whether reduced oxytocin levels are secondary to decreased oestrogen availability in the CNS remains unclear. Interestingly, oestrogen has been shown to regulate the gene expression of both oxytocin and its receptor [8]. It has been suggested that oxytocin administration may prevent migraine attacks [142]. The molecular mechanisms underlying oxytocin’s effects in migraine remain uncertain but are hypothesised to involve decreased excitability of TGVS neurons [145]. The localisation of oxytocin receptors in pain-relevant regions of both the CNS and PNS suggests multiple sites of action for its analgesic effects [39]. Notably, oestrogen may increase oxytocin levels, potentially reducing TGVS excitability [57]. Trigeminal neurons expressing oxytocin receptors may also contain CGRP, indicating that oxytocin could attenuate CGRP signalling and contribute to the anti-migraine effects of gepants and mAbs.

While the oxytocin theory of migraine is still developing, current evidence strongly supports a link between oxytocin and migraine pathophysiology.

## Vasopressin and its receptors

AVP is primarily known for its role in increasing tubular reabsorption and exerting antidiuretic effects. This mechanism, combined with its direct vasoconstrictive properties, contributes to elevated arterial blood pressure. AVP metabolism and bioavailability increase in response to stress and pain and have been associated with migraine pathophysiology, particularly with fluid retention early in attacks [154]. Recent studies have shown that AVP-containing nerve fibres are widely distributed throughout the CNS [38], including regions implicated in migraine such as the supraoptic nucleus, optic chiasm, and anterior hypothalamic area. These fibres also project to various brainstem centres, notably the TNC.

The action of AVP is mediated by three identified G-protein-coupled receptors: V1aR, V1bR, and V2R. V1aR is expressed on multiple cell types, including vascular smooth muscle, liver, platelets, brain, and spinal cord. V1bR is found in pancreatic islets and is widely expressed in the CNS [38]. In the brain, V1aR and V1bR are present in the cytoplasm of neurons within regions such as the hypothalamus, pons, and cerebellum. V1bR expression is more extensive than V1aR, occurring in the cortex, hippocampus (CA1 and CA3), brainstem, and inferior olive (IO). Several migraine-related regions, including the cortex, hippocampus, thalamus, hypothalamus, and cerebellum, express V1bR.

A particularly interesting and novel finding is the presence of AVP receptors (V1aR and V2R) in the TG [38]. In the TG, 75–85% of neurons exhibit immunoreactivity for V1aR and V1bR, while V2R is primarily expressed in satellite glial cells (SGCs). Double immunohistochemistry revealed that V1aR and V1bR co-localise with CGRP in the cytoplasm of small and medium-sized TG neurons. Additional staining demonstrated V1bR co-localisation with CASPR, a membrane protein expressed at the nodes of Ranvier of myelinated Aδ-fibres within the ganglion. V1bR expression was specifically localised to the paranodal region of myelinated trigeminal axons.

These findings suggest that part of the anti-migraine effect attributed to oxytocin may be mediated via V1bR, although further research is required to clarify this mechanism. The role of AVP and its receptors in migraine remains elusive, but may be linked to clinical features such as emesis, vomiting, hypovolaemia, and nausea.

## How can hypothalamic neuropeptides modify pain in migraine?

The role of oxytocin in migraine modulation remains a subject of debate. Oxytocin’s therapeutic effects in migraine are complex and involve widespread actions throughout the nervous system, including primary sensory neurons, the spinal cord, and multiple brain regions associated with pain processing and modulation [152]. Oxytocin exerts its pain-inhibitory effects both at the level of primary afferent fibres and within the CNS. Oestrogen can trigger oxytocin release and enhance its function by upregulating oxytocin receptors.

Within the CNS, oxytocin suppresses nociceptive sensitivity and alleviates headache pain by binding to oxytocin receptors in the TNC in the brainstem [155–159]. Preclinical studies indicate that oxytocin receptors are specifically expressed in neural tissue within the TG. However, the dura mater and cranial arteries do not express oxytocin receptor mRNA or protein, suggesting that oxytocin may directly modulate nociceptive signalling at the level of TG neurons [39, 41, 160].

Oxytocin may also engage pain-modulatory pathways in the CNS, including the TNC in the brainstem, midbrain, thalamus, and cortical pain-processing regions. Animal studies have identified two major oxytocinergic pathways that mediate analgesia: (i) projections from the PVN to the spinal dorsal horn via the midbrain periaqueductal grey (PAG), and (ii) projections from the SON to central pain-modulation circuits [156, 157].

Descending projections from the PVN to the spinal dorsal horn are considered a key mechanism of oxytocin-induced analgesia. PVN activation releases oxytocin into the dorsal horn, where it binds to receptors in laminae I–II and deeper layers [158, 159]. This oxytocin release produces an overall antinociceptive effect and pain inhibition. Oxytocin activates inhibitory GABAergic interneurons in the deep dorsal horn and excitatory glutamatergic interneurons in laminae I–II. PVN stimulation or oxytocin infusion also reduces activation of Aδ- and C-fibres in dorsal horn neurons, thereby inhibiting nociceptive input at the level of nociceptor terminals [156, 157].

Oxytocin appears to exert a complex effect on somatosensory transmission in the spinal dorsal horn. The precise mechanisms by which oxytocin modulates spinal interneuron subtypes to achieve net pain inhibition remain unclear but likely involve a combination of inhibitory and disinhibitory processes within spinal cord circuitry.

The SON in the hypothalamus is another important source of oxytocin-mediated analgesia [39]. Like the PVN, activation of SON neurons increases nociceptive thresholds via oxytocin release in several midbrain and brainstem regions involved in endogenous pain modulation, including the PAG, rostral ventromedial medulla (RVM), and the spinal dorsal horn. It is hypothesised that oxytocin receptors at these pain-modulation sites mediate oxytocin’s analgesic effects; however, the precise receptor mechanisms remain to be determined.

Oestrogen may regulate oxytocin release and the expression of oxytocin receptors. A recent theory proposes that menstrual-related migraine is associated with a decline in both oestrogen and oxytocin during menstruation [8]. Whether reduced oxytocin concentrations are secondary to diminished oestrogen availability in the CNS is not yet known.

Case reports have shown that intravenous oxytocin can provide analgesia and migraine relief [135, 136, 152], presumably at the level of the TG. Oxytocin receptor mRNA and protein are expressed in nociceptive C-fibres and Aδ-fibres in the adult rat TG [40]. Oxytocin may dose-dependently block CGRP release from trigeminal afferent neurons innervating the dura mater in vitro [40]. CGRP is critical for the pathogenesis of chronic migraine, suggesting that oxytocin receptor activation on trigeminal nociceptive neurons could be a key mechanism for reducing headache intensity and frequency following oxytocin administration.

Overall, preclinical studies indicate that oxytocin exerts a key anti-CGRP and anti-inflammatory role within the TG, which may reduce headache intensity and frequency when administered. However, the anti-migraine effects of oxytocin are more pronounced when the hormone can cross or bypass the BBB to reach the brain. Thus, increasing oxytocin concentrations in both the PNS and CNS may alleviate headache pain, and its association with minimal side effects makes it an appealing therapeutic candidate.

The main challenges are that systemic infusion of oxytocin is clinically impractical for widespread use, and its delivery to the CNS is hindered by its short plasma half-life (5–12 min) [17].

As an alternative, intranasal delivery of oxytocin offers a promising approach. Its primary advantage lies in the ability of oxytocin molecules to bypass the BBB, entering the trigeminal nerve via the nasal mucosa. Preclinical studies using radio-iodinated oxytocin have shown that nasally applied oxytocin concentrates in the trigeminal nerve and ganglia and deposits in various brain regions. Nevertheless, questions remain regarding the bioavailability of oxytocin in the brain following nasal administration.

## Conclusions

Migraine has for decades been observed to be more prevalent in women than in men. Since boys and girls show similar levels of migraine and genetic studies have not demonstrated a clear link, the search for a hormonal explanation is obvious. The early work by Somerville in the 1970s on the administration of oestrogen and progesterone to migraine patients led to the well-known “*oestrogen drop hypothesis*” in menstrual migraine [161], researchers have sought to address the question: *how might a decline in hormone levels be linked to the role of CGRP in migraine?* [8].

We have focused on sex hormones and two hypothalamic hormones that are most typical. The focus has been on the TGVS since this is currently most relevant in treatment of pain associated with migraine attacks. Future work will include the CNS regions hypothalamus and TNC since there are key regions in the initiation of migraine attacks [162]. The circulating hormones have access to the fibres and neurons of the TGVS since this region lacks BBB. There is a rich expression of receptors for estrogen, progesterone, testosterone, oxytocin and AVP in various parts of the TGVS suggesting multiple sites of interactions. The vascular receptors show in human middle meningeal artery moderate vasomotor responses with opposite directions (relaxation and contraction). The hormone receptors appear differentially in fibres, neurons and SGCs, which makes conclusions difficult. Stimulated release experiments from the TGVS did not reveal a clear-cut acute role of the hormones, since depolarization or capsaicin stimulated release of CGRP were not modified.

The evidence reviewed here indicates that fluctuations in oestrogen and progesterone remain the dominant hormonal drivers of menstrual-related vulnerability to migraine within the TGVS, while testosterone may exert a modest protective, antinociceptive influence. In parallel, the emerging roles of oxytocin and AVP, both strongly expressed in migraine-relevant CNS regions and the TGVS [38, 40], highlight that hypothalamic peptides also contribute to migraine susceptibility, particularly during the perimenstrual decline in circulating hormone levels. These combined findings support a model in which coordinated changes across sex steroids and hypothalamic neuropeptides dynamically modulate CGRP-related signalling, shaping individual sensitivity to migraine attacks.

A clearer hypothesis has emerged in a recent study examined how sex hormones regulate CGRP-related gene expression in the TG, with particular focus on RAMP1 [65]. The findings identify RAMP1 as a key mediator connecting hormonal fluctuations during the female cycle to CGRP signalling in the TG. Hormone-dependent changes in gene expression were sex-specific and disrupted in *RAMP1* knockout mice, supporting its role in migraine susceptibility. These results provide mechanistic insight into hormonal migraine and suggest that both acute hormone signalling and long-term epigenetic regulation influence individual sensitivity to CGRP-based therapies.

For the first time, there is clear evidence that oestrogen can regulate two critical components of CGRP signalling within the TGVS: the gene encoding CGRP and the gene expressing RAMP1. This discovery may offer clues for developing strategies to modulate hormonal influences in migraine pathophysiology and to design personalised therapies aimed at reducing the burden of menstrual-related migraine.

## Data Availability

The datasets generated during and/or analysed during the current study are available from the corresponding author on reasonable request.

## Abbreviations

BBB: Blood-brain barrier
CGRP: Calcitonin gene-related protein
CLR: Calcitonin-like receptor
TRPV1: Capsaicin receptor
CNS: Central Nervous System
CSD: Cortical spreading depression
Erα: Estrogen receptor alpha
Erβ: Estrogen receptor beta
GWAS: Genome-wide association study
GBD: Global Burden of Disease Study
GBDS: Global Burden of Disease Study
IHDC: International Headache Diagnostic Classification
MHT: Menopausal hormone therapy
MRT: Menopause replacement therapy
OT: Oxytocin
OTR: Oxytocin receptor
PVN: Paraventricular nucleus
PAG: Periaqueductal grey matter
PNS: Peripheral nervous system
PACAP: Pituitary adenylate cyclase-activating polypeptide
GPER: Protein-coupled estrogen receptor 1
RAMP1: Receptor activity-modifying protein 1
RVM: Rostral ventromedial medulla
Sp5: Spinal trigeminal tract
SCN: Suprachiasmatic nucleus
SON: Supraoptic nucleus
TWAS: Transcriptome-Wide Association Studies
TG: Trigeminal ganglion
TNC: Trigeminal nucleus caudalis
TGVS: Trigeminovascular system
AVP: Vasopressin
V1bR: Vasopressin receptor 1b
YLD: Years lived with disability

## References

[1] IHDC (2018) Headache classification committee of the international headache society (IHS) the international classification of headache disorders (IHDC), 3rd edition. Cephalalgia 38(1):1–211 10.1177/0333102417738202 29368949

[2] Lipton RB, Bigal ME, Diamond M, Freitag F, Reed ML, Stewart WF et al (2007) Migraine prevalence, disease burden, and the need for preventive therapy. Neurology 68(5):343–349 17261680 10.1212/01.wnl.0000252808.97649.21

[3] Collaborators GBDH (2018) Global, regional, and National burden of migraine and tension-type headache, 1990–2016: a systematic analysis for the global burden of disease study 2016. Lancet Neurol 17(11):954–976 30353868 10.1016/S1474-4422(18)30322-3 PMC6191530

[4] Vetvik KG, MacGregor EA (2017) Sex differences in the epidemiology, clinical features, and pathophysiology of migraine. Lancet Neurol 16(1):76–87 27836433 10.1016/S1474-4422(16)30293-9

[5] MacGregor EA (2020) Menstrual and perimenopausal migraine: A narrative review. Maturitas 142:24–30 33158484 10.1016/j.maturitas.2020.07.005

[6] Maddahi A, Warfvinge K, Holm A, Edvinsson JCA, Reducha PV, Kazantzi S et al (2023) Progesterone distribution in the trigeminal system and its role to modulate sensory neurotransmission: influence of sex. J Headache Pain 24(1):154 37957603 10.1186/s10194-023-01687-x PMC10644471

[7] Faubion SS, Batur P, Calhoun AH (2018) Migraine throughout the female reproductive life cycle. Mayo Clin Proc 93(5):639–645 10.1016/j.mayocp.2017.11.027 29728203

[8] Krause DN, Warfvinge K, Haanes KA, Edvinsson L (2021) Hormonal influences in migraine - interactions of oestrogen, Oxytocin and CGRP. Nat Rev Neurol 17(10):621–633 34545218 10.1038/s41582-021-00544-2

[9] Vetvik KG, MacGregor EA (2021) Menstrual migraine: a distinct disorder needing greater recognition. Lancet Neurol 20(4):304–315 33600767 10.1016/S1474-4422(20)30482-8

[10] Grangeon L, Lange KS, Waliszewska-Prosol M, Onan D, Marschollek K, Wiels W et al (2023) Genetics of migraine: where are we now? J Headache Pain 24(1):12 36800925 10.1186/s10194-023-01547-8 PMC9940421

[11] Gormley P, Anttila V, Winsvold BS, Palta P, Esko T, Pers TH et al (2016) Meta-analysis of 375,000 individuals identifies 38 susceptibility loci for migraine. Nat Genet 48(8):856–866 27322543 10.1038/ng.3598 PMC5331903

[12] Hautakangas H, Winsvold BS, Ruotsalainen SE, Bjornsdottir G, Harder AVE, Kogelman LJA et al (2022) Genome-wide analysis of 102,084 migraine cases identifies 123 risk loci and subtype-specific risk alleles. Nat Genet 54(2):152–160 35115687 10.1038/s41588-021-00990-0 PMC8837554

[13] Ghaffar A, Nyholt DR (2023) Integrating eQTL and GWAS data characterises established and identifies novel migraine risk loci. Hum Genet 142(8):1113–1137 37245199 10.1007/s00439-023-02568-8 PMC10449685

[14] Happola P, Gormley P, Nuottamo ME, Artto V, Sumelahti ML, Nissila M et al (2022) Polygenic risk provides biological validity for the ICHD-3 criteria among Finnish migraine families. Cephalalgia 42(4–5):345–356 34648375 10.1177/03331024211045651 PMC8988286

[15] Peterlin BL, Gupta S, Ward TN, Macgregor A (2011) Sex matters: evaluating sex and gender in migraine and headache research. Headache 51(6):839–842 21631471 10.1111/j.1526-4610.2011.01900.x PMC3975603

[16] MacGregor EA, Hackshaw A (2004) Prevalence of migraine on each day of the natural menstrual cycle. Neurology 63(2):351–353 15277635 10.1212/01.wnl.0000133134.68143.2e

[17] Silberstein SD, Merriam GR (1993) Sex hormones and headache. J Pain Symptom Manage 8(2):98–114 8492007 10.1016/0885-3924(93)90107-7

[18] Somerville BW (1972) The influence of progesterone and estradiol upon migraine. Headache 12(3):93–102 5075463 10.1111/j.1526-4610.1972.hed1203093.x

[19] Diotel N, Charlier TD, Lefebvre d’Hellencourt C, Couret D, Trudeau VL, Nicolau JC et al (2018) Steroid Transport, local Synthesis, and signaling within the brain: roles in Neurogenesis, Neuroprotection, and sexual behaviors. Front Neurosci 12:84 29515356 10.3389/fnins.2018.00084 PMC5826223

[20] Pavlovic JM (2018) Evaluation and management of migraine in midlife women. Menopause 25(8):927–929 29787480 10.1097/GME.0000000000001104 PMC6527322

[21] MacGregor EA (2004) Oestrogen and attacks of migraine with and without aura. Lancet Neurol 3(6):354–361 15157850 10.1016/S1474-4422(04)00768-9

[22] Feigin VL, Vos T, Nichols E, Owolabi MO, Carroll WM, Dichgans M et al (2020) The global burden of neurological disorders: translating evidence into policy. Lancet Neurol 19(3):255–265 31813850 10.1016/S1474-4422(19)30411-9 PMC9945815

[23] Lipton RB, Munjal S, Alam A, Buse DC, Fanning KM, Reed ML et al (2018) Migraine in America symptoms and treatment (MAST) study: baseline study Methods, treatment Patterns, and gender differences. Headache 58(9):1408–1426 30341895 10.1111/head.13407

[24] Kruit MC, Launer LJ, Overbosch J, van Buchem MA, Ferrari MD (2009) Iron accumulation in deep brain nuclei in migraine: a population-based magnetic resonance imaging study. Cephalalgia 29(3):351–359 19025553 10.1111/j.1468-2982.2008.01723.x PMC3268125

[25] Bolay H, Ozge A, Saginc P, Orekici G, Uluduz D, Yalin O et al (2015) Gender influences headache characteristics with increasing age in migraine patients. Cephalalgia 35(9):792–800 25424708 10.1177/0333102414559735

[26] Somerville BW (1972) The role of estradiol withdrawal in the etiology of menstrual migraine. Neurology 22(4):355–365 5062827 10.1212/wnl.22.4.355

[27] Somerville BW (1972) A study of migraine in pregnancy. Neurology 22(8):824–828 4673410 10.1212/wnl.22.8.824

[28] Somerville BW (1975) Estrogen-withdrawal migraine. II. Attempted prophylaxis by continuous estradiol administration. Neurology 25(3):245–250 1167631 10.1212/wnl.25.3.245

[29] Somerville BW (1975) Estrogen-withdrawal migraine. I. Duration of exposure required and attempted prophylaxis by premenstrual Estrogen administration. Neurology 25(3):239–244 1167630 10.1212/wnl.25.3.239

[30] Ceriani CEJ, Silberstein SD (2023) Current and emerging pharmacotherapy for menstrual migraine: a narrative review. Expert Opin Pharmacother 24(5):617–627 36946205 10.1080/14656566.2023.2194487

[31] Martin VT (2009) Ovarian hormones and pain response: a review of clinical and basic science studies. Gend Med 6 Suppl 2:168 – 92. 10.1016/j.genm.2009.03.006 19406368

[32] Brandes JL (2006) The influence of Estrogen on migraine: a systematic review. JAMA 295(15):1824–1830 16622144 10.1001/jama.295.15.1824

[33] Borsook D, Erpelding N, Lebel A, Linnman C, Veggeberg R, Grant PE et al (2014) Sex and the migraine brain. Neurobiol Dis 68:200–214 24662368 10.1016/j.nbd.2014.03.008 PMC4171725

[34] Delaruelle Z, Ivanova TA, Khan S, Negro A, Ornello R, Raffaelli B et al (2018) Male and female sex hormones in primary headaches. J Headache Pain 19(1):117 30497379 10.1186/s10194-018-0922-7 PMC6755575

[35] Maddahi A, Warfvinge K, Holm A, Edvinsson JC, Reducha PV, Kazantzi S et al (2023) Progesterone distribution in the trigeminal system and its role to modulate sensory neurotransmission: influence of sex. J Headache Pain 24(1):154 37957603 10.1186/s10194-023-01687-x PMC10644471

[36] Schulte LH, Mehnert J, May A (2020) Longitudinal neuroimaging over 30 days: Temporal characteristics of migraine. Ann Neurol 87(4):646–651 32031707 10.1002/ana.25697

[37] Schulte LH, May A (2016) The migraine generator revisited: continuous scanning of the migraine cycle over 30 days and three spontaneous attacks. Brain 139(Pt 7):1987–1993 27190019 10.1093/brain/aww097

[38] Maddahi A, Edvinsson L, Warfvinge K (2022) Expression of vasopressin and its receptors in migraine-related regions in CNS and the trigeminal system: influence of sex. J Headache Pain 23(1):152 36456902 10.1186/s10194-022-01524-7 PMC9713967

[39] Warfvinge K, Krause D, Edvinsson L (2020) The distribution of Oxytocin and the Oxytocin receptor in rat brain: relation to regions active in migraine. J Headache Pain 21(1):10 32028899 10.1186/s10194-020-1079-8 PMC7006173

[40] Warfvinge K, Krause DN, Maddahi A, Grell AS, Edvinsson JC, Haanes KA et al (2020) Oxytocin as a regulatory neuropeptide in the trigeminovascular system: Localization, expression and function of Oxytocin and Oxytocin receptors. Cephalalgia 40(12):1283–1295 32486908 10.1177/0333102420929027

[41] Warfvinge K, Krause DN, Maddahi A, Edvinsson JCA, Edvinsson L, Haanes KA (2020) Estrogen receptors alpha, beta and GPER in the CNS and trigeminal system - molecular and functional aspects. J Headache Pain 21(1):131 33167864 10.1186/s10194-020-01197-0 PMC7653779

[42] Edvinsson JCA, Vigano A, Alekseeva A, Alieva E, Arruda R, De Luca C et al (2020) The fifth cranial nerve in headaches. J Headache Pain 21(1):65 32503421 10.1186/s10194-020-01134-1 PMC7275328

[43] Edvinsson L, Uddman R (2005) Neurobiology in primary headaches. Brain Res Brain Res Rev 48(3):438–456 15914251 10.1016/j.brainresrev.2004.09.007

[44] Edvinsson L, Haanes KA, Warfvinge K, Krause DN (2018) CGRP as the target of new migraine therapies - successful translation from bench to clinic. Nat Rev Neurol 14(6):338–350 29691490 10.1038/s41582-018-0003-1

[45] Raffaelli B, Storch E, Overeem LH, Terhart M, Fitzek MP, Lange KS et al (2023) Sex hormones and calcitonin Gene-Related peptide in women with migraine: A Cross-sectional, matched cohort study. Neurology 100(17):e1825–e35 36813730 10.1212/WNL.0000000000207114 PMC10136010

[46] Goadsby PJ, Edvinsson L (1993) The trigeminovascular system and migraine: studies characterizing cerebrovascular and neuropeptide changes seen in humans and cats. Ann Neurol 33(1):48–56 8388188 10.1002/ana.410330109

[47] Goadsby PJ, Edvinsson L, Ekman R (1990) Vasoactive peptide release in the extracerebral circulation of humans during migraine headache. Ann Neurol 28(2):183–187 1699472 10.1002/ana.410280213

[48] Puledda F, Sacco S, Diener H-C, Ashina M, Al-Khazali HM, Ashina S et al (2024) International headache society global practice recommendations for the acute Pharmacological treatment of migraine. Cephalalgia 44(8):03331024241252666 10.1177/03331024241252666 39133176

[49] Sacco S, Amin FM, Ashina M, Bendtsen L, Deligianni CI, Gil-Gouveia R et al (2022) European headache federation guideline on the use of monoclonal antibodies targeting the calcitonin gene related peptide pathway for migraine prevention–2022 update. J Headache Pain 23(1):67 35690723 10.1186/s10194-022-01431-x PMC9188162

[50] Cohen F, Yuan H, Silberstein SD (2022) Calcitonin gene-related peptide (CGRP)-targeted monoclonal antibodies and antagonists in migraine: current evidence and rationale. BioDrugs 36(3):341–58 10.1007/s40259-022-00530-0 PMC9043885 35476215

[51] Bolay H, Berman NE, Akcali D (2011) Sex-related differences in animal models of migraine headache. Headache 51(6):891–904 21631475 10.1111/j.1526-4610.2011.01903.x

[52] Edvinsson L, Ekman R, Jansen I, Ottosson A, Uddman R (1987) Peptide-containing nerve fibers in human cerebral arteries: immunocytochemistry, radioimmunoassay, and in vitro Pharmacology. Ann Neurol 21(5):431–437 2438992 10.1002/ana.410210503

[53] Straube A, Andreou A (2019) Primary headaches during lifespan. J Headache Pain 20:1–14 30961531 10.1186/s10194-019-0985-0 PMC6734460

[54] Martin VT, Behbehani M (2006) Ovarian hormones and migraine headache: Understanding mechanisms and pathogenesis–part I. Headache 46(1):3–23 16412147 10.1111/j.1526-4610.2006.00309.x

[55] Martin VT, Behbehani M (2006) Ovarian hormones and migraine headache: Understanding mechanisms and pathogenesis–part 2. Headache 46(3):365–386 16618254 10.1111/j.1526-4610.2006.00370.x

[56] Neri I, Granella F, Nappi R, Manzoni GC, Facchinetti F, Genazzani AR (1993) Characteristics of headache at menopause: a clinico-epidemiologic study. Maturitas 17(1):31–37 8412841 10.1016/0378-5122(93)90121-w

[57] Bharadwaj VN, Porreca F, Cowan RP, Kori S, Silberstein SD, Yeomans DC (2021) A new hypothesis linking Oxytocin to menstrual migraine. Headache 61(7):1051–1059 34125955 10.1111/head.14152

[58] Martin VT (2008) New theories in the pathogenesis of menstrual migraine. Curr Pain Headache Rep 12(6):453–462 18973740 10.1007/s11916-008-0077-3

[59] Berman NE, Puri V, Chandrala S, Puri S, Macgregor R, Liverman CS et al (2006) Serotonin in trigeminal ganglia of female rodents: relevance to menstrual migraine. Headache: J Head Face Pain 46(8):1230–1245 10.1111/j.1526-4610.2006.00528.x 16942467

[60] KLAIBER EL, KOBAYASHI Y, BROVERMAN DM, HALL F (1971) Plasma monoamine oxidase activity in regularly menstruating women and in amenorrheic women receiving Cyclic treatment with estrogens and a progestin. J Clin Endocrinol Metabolism 33(4):630–638 10.1210/jcem-33-4-630 4328587

[61] Edvinsson JC, Grubor I, Maddahi A, Edvinsson L (2024) Male-female comparison of vasomotor effects of Circulating hormones in human intracranial arteries. J Headache Pain 25(1):1–13 39663536 10.1186/s10194-024-01933-w PMC11633024

[62] Welch KM, Brandes JL, Berman NE (2006) Mismatch in how oestrogen modulates molecular and neuronal function May explain menstrual migraine. Neurol Sci 27(Suppl 2):S190–S192 16688628 10.1007/s10072-006-0599-6

[63] Sarajari S, Oblinger MM (2010) Estrogen effects on pain sensitivity and neuropeptide expression in rat sensory neurons. Exp Neurol 224(1):163–169 20303952 10.1016/j.expneurol.2010.03.006 PMC2885587

[64] Negro A, Delaruelle Z, Ivanova TA, Khan S, Ornello R, Raffaelli B et al (2017) Headache and pregnancy: a systematic review. J Headache Pain 18(1):106 29052046 10.1186/s10194-017-0816-0 PMC5648730

[65] Holm A, Edvinsson JCA, Krause DN, Edvinsson L (2025) RAMP1-dependent hormonal regulation of CGRP and its receptor in the trigeminal ganglion. J Headache Pain 26(1):142 40528180 10.1186/s10194-025-02071-7 PMC12172354

[66] Khandelwal S, Meeta M, Tanvir T (2022) Menopause hormone therapy, migraines, and thromboembolism. Best Pract Res Clin Obstet Gynaecol 81:31–44 34974967 10.1016/j.bpobgyn.2021.11.011

[67] MacGregor EA (2007) Migraine, the menopause and hormone replacement therapy: a clinical review. J Fam Plann Reprod Health Care 33(4):245–249 17925104 10.1783/147118907782101986

[68] Sacco S, Ricci S, Degan D, Carolei A (2012) Migraine in women: the role of hormones and their impact on vascular diseases. J Headache Pain 13(3):177–189 22367631 10.1007/s10194-012-0424-y PMC3311830

[69] Wang H, Ward A, Morris J (1995) Oestradiol acutely stimulates exocytosis of Oxytocin and vasopressin from dendrites and Somata of hypothalamic magnocellular neurons. Neuroscience 68(4):1179–1188 8544991 10.1016/0306-4522(95)00186-m

[70] Somponpun S, Sladek CD (2002) Role of Estrogen receptor-β in regulation of vasopressin and Oxytocin release in vitro. Endocrinology 143(8):2899–2904 12130554 10.1210/endo.143.8.8946

[71] Acevedo-Rodriguez A, Mani SK, Handa RJ (2015) Oxytocin and Estrogen receptor β in the brain: an overview. Front Endocrinol 6:160 10.3389/fendo.2015.00160 PMC4606117 26528239

[72] Storch E, Overeem LH, Terhart M, Fitzek MP, Lange KS, Reuter U et al (2024) PACAP-38 and sex hormones in women with migraine: exploratory analysis of a cross-sectional, matched cohort study. J Headache Pain 25(1):98 38858641 10.1186/s10194-024-01804-4 PMC11165852

[73] Marcus DA, Scharff L, Turk D (1999) Longitudinal prospective study of headache during pregnancy and postpartum. Headache 39(9):625–632 11279958 10.1046/j.1526-4610.1999.3909625.x

[74] Valdemarsson S, Edvinsson L, Hedner P, Ekman R (1990) Hormonal influence on calcitonin gene-related peptide in man: effects of sex difference and contraceptive pills. Scand J Clin Lab Invest 50(4):385–388 2392651 10.3109/00365519009091595

[75] Maggioni F, Alessi C, Maggino T, Zanchin G (1997) Headache during pregnancy. Cephalalgia 17(7):765–769 9399007 10.1046/j.1468-2982.1997.1707765.x

[76] Granella F, Sances G, Pucci E, Nappi RE, Ghiotto N, Napp G (2000) Migraine with aura and reproductive life events: a case control study. Cephalalgia 20(8):701–707 11167898 10.1111/j.1468-2982.2000.00112.x

[77] Pardutz A, Multon S, Malgrange B, Parducz A, Vecsei L, Schoenen J (2002) Effect of systemic nitroglycerin on CGRP and 5-HT afferents to rat caudal spinal trigeminal nucleus and its modulation by Estrogen. Eur J Neurosci 15(11):1803–1809 12081660 10.1046/j.1460-9568.2002.02031.x

[78] Ibrahimi K, Vermeersch S, Frederiks P, Geldhof V, Draulans C, Buntinx L et al (2017) The influence of migraine and female hormones on capsaicin-induced dermal blood flow. Cephalalgia 37(12):1164–1172 27687880 10.1177/0333102416668659

[79] Miller VM, Duckles SP (2008) Vascular actions of estrogens: functional implications. Pharmacol Rev 60(2):210–241 18579753 10.1124/pr.107.08002 PMC2637768

[80] Chen P, Li B, Ou-Yang L (2022) Role of Estrogen receptors in health and disease. Front Endocrinol (Lausanne) 13:839005 36060947 10.3389/fendo.2022.839005 PMC9433670

[81] De Lignieres B, Vincens M, Mauvais-Jarvis P, Mas J, Touboul P, Bousser M (1986) Prevention of menstrual migraine by percutaneous oestradiol. Br Med J (Clinical Res ed) 293(6561):1540 10.1136/bmj.293.6561.1540 PMC1342315 3099950

[82] Dennerstein L, Morse C, Burrows G, Oats J, Brown J, Smith M (1988) Menstrual migraine: a double-blind trial of percutaneous estradiol. Gynecol Endocrinol 2(2):113–120 3055819 10.3109/09513598809023619

[83] Warfvinge K, Krause DN, Maddahi A, Edvinsson JC, Edvinsson L, Haanes KA (2020) Estrogen receptors α, β and GPER in the CNS and trigeminal system-molecular and functional aspects. J Headache Pain 21(1):131 33167864 10.1186/s10194-020-01197-0 PMC7653779

[84] Luo J, Liu D (2020) Does GPER really function as a G Protein-Coupled Estrogen receptor in vivo? Front Endocrinol (Lausanne) 11:148 32296387 10.3389/fendo.2020.00148 PMC7137379

[85] Taraborrelli S (2015) Physiology, production and action of progesterone. Acta Obstet Gynecol Scand 94:8–16 26358238 10.1111/aogs.12771

[86] Conneely OM, Mulac-Jericevic B, DeMayo F, Lydon JP, O Malley BW (2002) Reproductive functions of progesterone receptors. Recent Prog Horm Res 57:339–356 10.1210/rp.57.1.339 12017551

[87] Balfour W, Comline RS, Short RV (1957) Secretion of progesterone by the adrenal gland. Nature 180(4600):1480–1481 13493572 10.1038/1801480a0

[88] De Nicola AF, Garay LI, Meyer M, Guennoun R, Sitruk-Ware R, Schumacher M et al (2018) Neurosteroidogenesis and progesterone anti-inflammatory/neuroprotective effects. J Neuroendocrinol 30(2) 10.1111/jne.12502 28675779

[89] Wegiel J, Flory M, Kuchna I, Nowicki K, Ma SY, Imaki H et al (2015) Neuronal nucleus and cytoplasm volume deficit in children with autism and volume increase in adolescents and adults. Acta Neuropathol Commun 3(1):2 25595448 10.1186/s40478-015-0183-5 PMC4302585

[90] Landau RL, Bergenstal DM, Lugibihl K, Kascht ME (1955) The metabolic effects of progesterone in man. J Clin Endocrinol Metab 15(10):1194–1215 13263410 10.1210/jcem-15-10-1194

[91] Luoma JI, Stern CM, Mermelstein PG (2012) Progesterone Inhibition of neuronal calcium signaling underlies aspects of progesterone-mediated neuroprotection. J Steroid Biochem Mol Biol 131(1–2):30–36 22101209 10.1016/j.jsbmb.2011.11.002 PMC3303940

[92] Schaeffer V, Meyer L, Patte-Mensah C, Mensah-Nyagan AG (2010) Progress in dorsal root ganglion neurosteroidogenic activity: basic evidence and pathophysiological correlation. Prog Neurobiol 92(1):33–41 20438798 10.1016/j.pneurobio.2010.04.009

[93] Limmroth V, Lee WS, Moskowitz MA (1996) GABAA-receptor‐mediated effects of progesterone, its ring‐A‐reduced metabolites and synthetic neuroactive steroids on neurogenic oedema in the rat meninges. Br J Pharmacol 117(1):99–104 8825349 10.1111/j.1476-5381.1996.tb15160.x PMC1909356

[94] Singh M (2001) Ovarian hormones elicit phosphorylation of Akt and extracellular-signal regulated kinase in explants of the cerebral cortex. Endocrine 14(3):407–415 11444439 10.1385/ENDO:14:3:407

[95] Davis SR, Wahlin-Jacobsen S (2015) Testosterone in women—the clinical significance. Lancet Diabetes Endocrinol 3(12):980–992 26358173 10.1016/S2213-8587(15)00284-3

[96] Atukorala KR, Silva W, Amarasiri L, Fernando D (2022) Changes in serum testosterone during the menstrual cycle—an integrative systematic review of published literature. Gynecol Reprod Endocrinol Metab 3:9–20

[97] Pringsheim T, Gooren L (2004) Migraine prevalence in male to female transsexuals on hormone therapy. Neurology 63(3):593–594 15304612 10.1212/01.wnl.0000130338.62037.cc

[98] Bartley EJ, Palit S, Kuhn BL, Kerr KL, Terry EL, DelVentura JL et al (2015) Nociceptive processing in women with premenstrual dysphoric disorder (PMDD): the role of menstrual phase and sex hormones. Clin J Pain 31(4):304–314 24810651 10.1097/AJP.0000000000000112

[99] Bartley EJ, Palit S, Kuhn BL, Kerr KL, Terry EL, DelVentura JL et al (2015) Natural variation in testosterone is associated with hypoalgesia in healthy women. Clin J Pain 31(8):730–739 25185874 10.1097/AJP.0000000000000153

[100] Choi JC, Chung MI, Lee YD (2012) Modulation of pain sensation by stress-related testosterone and cortisol. Anaesthesia 67(10):1146–1151 22804789 10.1111/j.1365-2044.2012.07267.x

[101] Choi JC, Park YH, Park SK, Lee JS, Kim J, Choi JI et al (2017) Testosterone effects on pain and brain activation patterns. Acta Anaesthesiol Scand 61(6):668–675 28573655 10.1111/aas.12908

[102] Ceccarelli I, Scaramuzzino A, Massafra C, Aloisi AM (2003) The behavioral and neuronal effects induced by repetitive nociceptive stimulation are affected by gonadal hormones in male rats. Pain 104(1–2):35–47 12855312 10.1016/s0304-3959(02)00460-8

[103] Stoffel EC, Ulibarri CM, Craft RM (2003) Gonadal steroid hormone modulation of nociception, morphine antinociception and reproductive indices in male and female rats. Pain 103(3):285–302 12791435 10.1016/s0304-3959(02)00457-8 PMC1420267

[104] Aloisi AM, Ceccarelli I, Fiorenzani P, De Padova AM, Massafra C (2004) Testosterone affects formalin-induced responses differently in male and female rats. Neurosci Lett 361(1–3):262–264 15135943 10.1016/j.neulet.2003.12.023

[105] Gaumond I, Arsenault P, Marchand S (2005) Specificity of female and male sex hormones on excitatory and inhibitory phases of formalin-induced nociceptive responses. Brain Res 1052(1):105–111 16005855 10.1016/j.brainres.2005.06.011

[106] Basaria S, Travison TG, Alford D, Knapp PE, Teeter K, Cahalan C et al (2015) Effects of testosterone replacement in men with opioid-induced androgen deficiency: a randomized controlled trial. Pain 156(2):280–288 25599449 10.1097/01.j.pain.0000460308.86819.aa PMC6036339

[107] Grande G, Labruijere S, Haanes KA, MaassenVanDenBrink A, Edvinsson L (2014) Comparison of the vasodilator responses of isolated human and rat middle meningeal arteries to migraine related compounds. J Headache Pain 15(1):22 24754925 10.1186/1129-2377-15-22 PMC4011837

[108] Edvinsson JCA, Grell AS, Warfvinge K, Sheykhzade M, Edvinsson L, Haanes KA (2020) Differences in pituitary adenylate cyclase-activating peptide and calcitonin gene-related peptide release in the trigeminovascular system. Cephalalgia 40(12):1296–1309 32486909 10.1177/0333102420929026

[109] Karsan N, Edvinsson L, Vecsei L, Goadsby PJ (2024) Pituitary cyclase-activating polypeptide targeted treatments for the treatment of primary headache disorders. Ann Clin Transl Neurol 11(7):1654–1668 38887982 10.1002/acn3.52119 PMC11251486

[110] Maddahi A, Edvinsson L, Warfvinge K (2022) Expression of vasopressin and its receptors in migraine-related regions in CNS and the trigeminal system: influence of sex. J Headache Pain 23(1):1–20 36456902 10.1186/s10194-022-01524-7 PMC9713967

[111] Charles A (2013) The evolution of a migraine attack - a review of recent evidence. Headache 53(2):413–419 23278169 10.1111/head.12026

[112] Dodick DW (2018) A phase-by‐phase review of migraine pathophysiology. Headache: J Head Face Pain 58:4–16 10.1111/head.13300 29697154

[113] Schulte LH, Allers A, May A (2017) Hypothalamus as a mediator of chronic migraine: evidence from high-resolution fMRI. Neurology 88(21):2011–2016 28446645 10.1212/WNL.0000000000003963

[114] Graham J, Wollf H (1938) MECHANISM OF MIGRAINE HEADACHE AND ACTION OF ERGOTAMINE TARTRATE. Arch NeurPsych 39(4):737–763

[115] Ashina M, Hansen JM, BO AD, Olesen J (2017) Human models of migraine - short-term pain for long-term gain. Nat Rev Neurol 13(12):713–724 28984313 10.1038/nrneurol.2017.137

[116] Olesen J (2024) Provocation of attacks to discover migraine signaling mechanisms and new drug targets: early history and future perspectives-a narrative review. J Headache Pain 25(1):105 38902612 10.1186/s10194-024-01796-1 PMC11188241

[117] Winsvold BS, Nelson CP, Malik R, Gormley P, Anttila V, Vander Heiden J et al (2015) Genetic analysis for a shared biological basis between migraine and coronary artery disease. Neurol Genet 1(1):e10 27066539 10.1212/NXG.0000000000000010 PMC4821079

[118] Dodick D, Edvinsson L, Makino T, Grisold W, Sakai F, Jensen R et al (2018) Vancouver declaration on global headache patient advocacy 2018. Cephalalgia 38(13):1899–1909 29882695 10.1177/0333102418781644

[119] May A, Burstein R (2019) Hypothalamic regulation of headache and migraine. Cephalalgia 39(13):1710–1719 31466456 10.1177/0333102419867280 PMC7164212

[120] Weiller C, May A, Limmroth V, Juptner M, Kaube H, Schayck RV et al (1995) Brain stem activation in spontaneous human migraine attacks. Nat Med 1(7):658–660 7585147 10.1038/nm0795-658

[121] Meylakh N, Marciszewski KK, Di Pietro F, Macefield VG, Macey PM, Henderson LA (2020) Altered regional cerebral blood flow and hypothalamic connectivity immediately prior to a migraine headache. Cephalalgia 40(5):448–460 32164427 10.1177/0333102420911623

[122] Maddahi A, Edvinsson L, Krause DN (2025) Expression of melatonin receptors in trigeminal and Sphenopalatine ganglia: potential targets for primary headache disorders 10.1186/s10194-025-02215-9 PMC12690866 41366729

[123] Bahra A, Matharu MS, Buchel C, Frackowiak RS, Goadsby PJ (2001) Brainstem activation specific to migraine headache. Lancet 357(9261):1016–1017 11293599 10.1016/s0140-6736(00)04250-1

[124] May A (2017) Understanding migraine as a cycling brain syndrome: reviewing the evidence from functional imaging. Neurol Sci 38(Suppl 1):125–130 28527054 10.1007/s10072-017-2866-0

[125] Moskowitz MA (2007) Genes, proteases, cortical spreading depression and migraine: impact on pathophysiology and treatment. Funct Neurol 22(3):133–136 17925161

[126] Mehnert J, Fischer-Schulte L, May A (2023) Aura phenomena do not initiate migraine attacks-Findings from neuroimaging. Headache 63(8):1040–1044 37449553 10.1111/head.14597

[127] Charles A (2023) Aura is a symptom of a migraine attack, not its cause. Headache 63(8):1029–1030 37665161 10.1111/head.14623

[128] May A (2024) Beyond aura: Understanding migraine as a cycling pan-sensory threshold disease. Wiley Online Library, pp 715–717 10.1111/head.14736 38779974

[129] Eftekhari S, Salvatore CA, Johansson S, Chen TB, Zeng Z, Edvinsson L (2015) Localization of CGRP, CGRP receptor, PACAP and glutamate in trigeminal ganglion. Relation to the blood-brain barrier. Brain Res 1600:93–109 25463029 10.1016/j.brainres.2014.11.031

[130] Lundblad C, Haanes KA, Grande G, Edvinsson L (2015) Experimental inflammation following dural application of complete freund’s adjuvant or inflammatory soup does not alter brain and trigeminal microvascular passage. J Headache Pain 16:91 26512021 10.1186/s10194-015-0575-8 PMC4627622

[131] Noseda R, Schain AJ, Melo-Carrillo A, Tien J, Stratton J, Mai F et al (2020) Fluorescently-labeled fremanezumab is distributed to sensory and autonomic ganglia and the dura but not to the brain of rats with uncompromised blood brain barrier. Cephalalgia 40(3):229–240 31856583 10.1177/0333102419896760 PMC7233263

[132] Edvinsson JC, Warfvinge K, Krause DN, Blixt FW, Sheykhzade M, Edvinsson L et al (2019) C-fibers May modulate adjacent Aδ-fibers through axon-axon CGRP signaling at nodes of Ranvier in the trigeminal system. J Headache Pain 20:1–10 31718551 10.1186/s10194-019-1055-3 PMC6852900

[133] Edvinsson L (2015) CGRP receptor antagonists and antibodies against CGRP and its receptor in migraine treatment. Br J Clin Pharmacol 80(2):193–199 25731075 10.1111/bcp.12618 PMC4541967

[134] Mikitsh JL, Chacko AM (2014) Pathways for small molecule delivery to the central nervous system across the blood-brain barrier. Perspect Medicin Chem 6:11–24 24963272 10.4137/PMC.S13384 PMC4064947

[135] Phillips WJ, Ostrovsky O, Galli RL, Dickey S (2006) Relief of acute migraine headache with intravenous oxytocin: report of two cases. J Pain Palliat Care Pharmacother 20(3):25–28 16931475

[136] Tzabazis A, Kori S, Mechanic J, Miller J, Pascual C, Manering N et al (2017) Oxytocin and migraine headache. Headache 57(Suppl 2):64–75 28485846 10.1111/head.13082

[137] Wang YL, Yuan Y, Yang J, Wang CH, Pan YJ, Lu L et al (2013) The interaction between the Oxytocin and pain modulation in headache patients. Neuropeptides 47(2):93–97 23375440 10.1016/j.npep.2012.12.003

[138] Murphy D, Waller S, Fairhall K, Carter DA, Robinson CA (1998) Regulation of the synthesis and secretion of vasopressin. Prog Brain Res 119:137–143 10074786 10.1016/s0079-6123(08)61567-8

[139] Jirikowski GF (2019) Diversity of central oxytocinergic projections. Cell Tissue Res 375(1):41–48 30498946 10.1007/s00441-018-2960-5

[140] Ludwig M (1998) Dendritic release of vasopressin and Oxytocin. J Neuroendocrinol 10(12):881–895 9870745 10.1046/j.1365-2826.1998.00279.x

[141] Szewczyk AK, Ulutas S, Akturk T, Al-Hassany L, Borner C, Cernigliaro F et al (2023) Prolactin and oxytocin: potential targets for migraine treatment. J Headache Pain 24(1):31 36967387 10.1186/s10194-023-01557-6 PMC10041814

[142] Tzabazis A, Mechanic J, Miller J, Klukinov M, Pascual C, Manering N et al (2016) Oxytocin receptor: expression in the trigeminal nociceptive system and potential role in the treatment of headache disorders. Cephalalgia 36(10):943–950 26590611 10.1177/0333102415618615

[143] Gimpl G, Fahrenholz F (2001) The Oxytocin receptor system: structure, function, and regulation. Physiol Rev 81(2):629–683 11274341 10.1152/physrev.2001.81.2.629

[144] Gong L, Gao F, Li J, Li J, Yu X, Ma X et al (2015) Oxytocin-induced membrane hyperpolarization in pain-sensitive dorsal root ganglia neurons mediated by Ca(2+)/nNOS/NO/KATP pathway. Neuroscience 289:417–28 10.1016/j.neuroscience.2014.12.058 25617653

[145] Kubo A, Shinoda M, Katagiri A, Takeda M, Suzuki T, Asaka J et al (2017) Oxytocin alleviates orofacial mechanical hypersensitivity associated with infraorbital nerve injury through vasopressin-1A receptors of the rat trigeminal ganglia. Pain 158(4):649–659 28072605 10.1097/j.pain.0000000000000808

[146] Garcia-Boll E, Martinez-Lorenzana G, Condes-Lara M, Gonzalez-Hernandez A (2018) Oxytocin inhibits the rat medullary dorsal Horn Sp5c/C1 nociceptive transmission through OT but not V(1A) receptors. Neuropharmacology 129:109–117 29169960 10.1016/j.neuropharm.2017.11.031

[147] Paloyelis Y, Krahe C, Maltezos S, Williams SC, Howard MA, Fotopoulou A (2016) The analgesic effect of oxytocin in humans: a double-blind, placebo-controlled cross-over study using laser-evoked potentials. J Neuroendocrinol 28(4) 10.1111/jne.12347 PMC5103211 26660859

[148] MacDonald E, Dadds MR, Brennan JL, Williams K, Levy F, Cauchi AJ (2011) A review of safety, side-effects and subjective reactions to intranasal Oxytocin in human research. Psychoneuroendocrinology 36(8):1114–1126 21429671 10.1016/j.psyneuen.2011.02.015

[149] Kuwabara Y, Takeda S, Mizuno M, Sakamoto S (1987) Oxytocin levels in maternal and fetal plasma, amniotic fluid, and neonatal plasma and urine. Arch Gynecol Obstet 241(1):13–23 3674982 10.1007/BF00931436

[150] Hoshiyama E, Tatsumoto M, Iwanami H, Saisu A, Watanabe H, Inaba N et al (2012) Postpartum migraines: a long-term prospective study. Intern Med 51(22):3119–3123 23154716 10.2169/internalmedicine.51.8542

[151] Bharadwaj VN, Tzabazis AZ, Klukinov M, Manering NA, Yeomans DC (2021) Intranasal administration for pain: oxytocin and other polypeptides. Pharmaceutics 13(7) 10.3390/pharmaceutics13071088 PMC8309171 34371778

[152] Rash JA, Aguirre-Camacho A, Campbell TS (2014) Oxytocin and pain: a systematic review and synthesis of findings. Clin J Pain 30(5):453–462 23887343 10.1097/AJP.0b013e31829f57df

[153] Iwasaki M, Lefevre A, Althammer F, Clauss Creusot E, Lapies O, Petitjean H et al (2023) An analgesic pathway from parvocellular Oxytocin neurons to the periaqueductal Gray in rats. Nat Commun 14(1):1066 36828816 10.1038/s41467-023-36641-7 PMC9958129

[154] Dalkara T, Nozari A, Moskowitz MA (2010) Migraine aura pathophysiology: the role of blood vessels and microembolisation. Lancet Neurol 9(3):309–317 20170844 10.1016/S1474-4422(09)70358-8 PMC2921876

[155] Miranda-Cardenas Y, Rojas-Piloni G, Martinez-Lorenzana G, Rodriguez-Jimenez J, Lopez-Hidalgo M, Freund-Mercier MJ et al (2006) Oxytocin and electrical stimulation of the paraventricular hypothalamic nucleus produce antinociceptive effects that are reversed by an Oxytocin antagonist. Pain 122(1–2):182–189 16527400 10.1016/j.pain.2006.01.029

[156] Yang J, Liang JY, Li P, Pan YJ, Qiu PY, Zhang J et al (2011) Oxytocin in the periaqueductal Gray participates in pain modulation in the rat by influencing endogenous opiate peptides. Peptides 32(6):1255–1261 21439337 10.1016/j.peptides.2011.03.007

[157] Yang J, Li P, Liang JY, Pan YJ, Yan XQ, Yan FL et al (2011) Oxytocin in the periaqueductal grey regulates nociception in the rat. Regul Pept 169(1–3):39–42 21545817 10.1016/j.regpep.2011.04.007

[158] Yang J, Yang Y, Chen JM, Liu WY, Wang CH, Lin BC (2007) Central Oxytocin enhances antinociception in the rat. Peptides 28(5):1113–1119 17420069 10.1016/j.peptides.2007.03.003

[159] Yang J, Yang Y, Chen JM, Liu WY, Lin BC (2008) Investigating the role of the hypothalamic supraoptic nucleus in nociception in the rat. Life Sci 82(3–4):166–173 18062994 10.1016/j.lfs.2007.10.023

[160] Swanson LW, McKellar S (1979) The distribution of oxytocin- and neurophysin-stained fibers in the spinal cord of the rat and monkey. J Comp Neurol 188(1):87–106 115910 10.1002/cne.901880108

[161] Somerville BW (1972) The role of estradiol withdrawal in the etiology of menstrual migraine. Neurology 22(4):355 5062827 10.1212/wnl.22.4.355

[162] Peng KP, May A (2025) Cycling sensitivity across migraine phases: A longitudinal case–control study. Eur J Pain 29(1):e4761 39578922 10.1002/ejp.4761 PMC11609900

[163] Liu Y, Broman J, Zhang M, Edvinsson L (2009) Brainstem and thalamic projections from a craniovascular sensory nervous centre in the rostral cervical spinal dorsal Horn of rats. Cephalalgia 29(9):935–948 19250290 10.1111/j.1468-2982.2008.01829.x

